# Hetero-bivalent nanobodies provide broad-spectrum protection against SARS-CoV-2 variants of concern including Omicron

**DOI:** 10.1038/s41422-022-00700-3

**Published:** 2022-07-29

**Authors:** Huan Ma, Xinghai Zhang, Peiyi Zheng, Peter H. Dube, Weihong Zeng, Shaohong Chen, Qingyu Cheng, Yunru Yang, Yan Wu, Junhui Zhou, Xiaowen Hu, Yan Xiang, Huajun Zhang, Sandra Chiu, Tengchuan Jin

**Affiliations:** 1 Department of Pulmonary and Critical Care Medicine, The First Affiliated Hospital of USTC, Division of Life Sciences and Medicine, University of Science and Technology of China, Hefei, Anhui China; 2 State Key Laboratory of Virology, Wuhan Institute of Virology, Center for Biosafety Mega-Science, Chinese Academy of Sciences, Wuhan, Hubei China; 3 Laboratory of Structural Immunology, CAS Key Laboratory of Innate Immunity and Chronic Disease, Hefei National Laboratory for Physical Sciences at Microscale, Division of Life Sciences and Medicine, University of Science and Technology of China, Hefei, Anhui China; 4 Department of Microbiology, Immunology and Molecular Genetics, University of Texas Health Science Center at San Antonio, San Antonio, TX USA; 5 University of Chinese Academy of Sciences, Beijing, China; 6 Institute of Health and Medicine, Hefei Comprehensive National Science Center, Hefei, Anhui China

**Keywords:** X-ray crystallography, Innate immunity

## Abstract

SARS-CoV-2 variants with adaptive mutations have continued to emerge, causing fresh waves of infection even amongst vaccinated population. The development of broad-spectrum antivirals is thus urgently needed. We previously developed two hetero-bivalent nanobodies (Nbs), aRBD-2-5 and aRBD-2-7, with potent neutralization activity against the wild-type (WT) Wuhan isolated SARS-CoV-2, by fusing aRBD-2 with aRBD-5 and aRBD-7, respectively. Here, we resolved the crystal structures of these Nbs in complex with the receptor-binding domain (RBD) of the spike protein, and found that aRBD-2 contacts with highly-conserved RBD residues and retains binding to the RBD of the Alpha, Beta, Gamma, Delta, Delta plus, Kappa, Lambda, Omicron BA.1, and BA.2 variants. In contrast, aRBD-5 and aRBD-7 bind to less-conserved RBD epitopes non-overlapping with the epitope of aRBD-2, and do not show apparent binding to the RBD of some variants. However, when fused with aRBD-2, they effectively enhance the overall binding affinity. Consistently, aRBD-2-5-Fc and aRBD-2-7-Fc potently neutralized all of the tested authentic or pseudotyped viruses, including WT, Alpha, Beta, Gamma, Delta, and Omicron BA.1, BA.1.1 and BA.2. Furthermore, aRBD-2-5-Fc provided prophylactic protection against the WT and mouse-adapted SARS-CoV-2 in mice, and conferred protection against the Omicron BA.1 variant in hamsters prophylactically and therapeutically, indicating that aRBD-2-5-Fc could potentially benefit the prevention and treatment of COVID-19 caused by the emerging variants of concern. Our strategy provides new solutions in the development of broad-spectrum therapeutic antibodies for COVID-19.

## Introduction

The pandemic caused by the severe acute respiratory syndrome coronavirus 2 (SARS-CoV-2) continues to threaten global health and economic development. The spread in global populations and adaptive evolution of SARS-CoV-2 have contributed to the emergence of variants of concern (VOCs), which have replaced the original virus and become dominant strains around the world.^1–5^ Although vaccines are considered the terminators of this epidemic, the accumulation of mutations in VOCs, especially in the Omicron variant, which carries more than 30 mutations in the spike protein, has weakened the efficacy of most approved vaccines designed against the original Wuhan isolate.^6–8^ Indeed, breakthrough infections with VOCs have been reported amongst fully-vaccinated populations in multiple regions of the world. Coupled with the insufficient vaccine response in immunocompromised individuals, vaccine shortages in low-income countries, and vaccine ineffectiveness,^9–11^ the development of effective prophylactic and therapeutic drugs to combat SARS-CoV-2 VOCs is essential.

In addition to vaccines for active immunization, SARS-CoV-2 neutralizing antibodies targeting the receptor binding domain (RBD) for passive immunization are another promising approach against COVID-19.^12,13^ However, most approved antibodies have no or greatly reduced activity in neutralizing the emerging VOCs, particularly the Omicron variant.^14–18^ With several advantages over conventional antibodies, variable fragments of heavy-chain-only antibodies (VHHs) derived from camelid, also called nanobodies (Nbs), are considered attractive alternatives to conventional antibodies.^19–21^ To date, a number of Nbs against SARS-CoV-2 RBD have been isolated from synthetic or immunized libraries.^22–45^ However, these Nbs also face the challenge posed by the VOCs, and might be ineffective or have reduced efficacy against the Omicron variant like the previously developed conventional antibodies.

Nbs consist of only one antigen-binding domain and are therefore easily designed into multimeric form to generate additional binding properties.^46^ We previously developed two hetero-bivalent Nbs, aRBD-2-5 and aRBD-2-7, by tandemly fusing monovalent aRBD-2 with aRBD-5 and aRBD-7, respectively, and demonstrated their potent neutralization activity against the wild-type (WT) SARS-CoV-2.^47^ In this study, we determined the structures of these Nbs in complex with the RBD, and showed that they could provide potent and broad-spectrum protection both in vitro and in vivo against VOCs, including the current circulating Omicron variant.

## Results

### aRBD-2 binds to a conserved RBM epitope and does not compete with aRBD-5 and aRBD-7

**Fig. 1:**
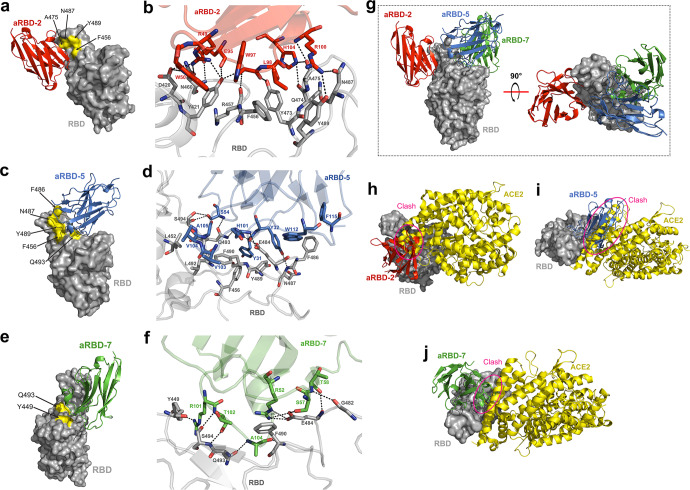
Structural analysis of aRBD-2, aRBD-5, and aRBD-7 binding to the WT SARS-CoV-2 RBD. **a–f** Three-dimensional structures of aRBD-2 (**a**, red), aRBD-5 (**c**, marine) and aRBD-7 (**e**, green) bound to the SARS-CoV-2 RBD (gray). The overlapped epitope residues between the Nbs and ACE2 on RBD are highlighted in yellow. The interacting residues are shown as sticks in the zoomed-in view of aRBD-2 (**b**), aRBD-5 (**d**), and aRBD-7 (**f**) in complex with RBD, and the hydrogen bonds and salt bridges between the Nbs and RBD are shown as black dotted lines. **g** The superimposition of the structures of aRBD-2, aRBD-5 and aRBD-7 bound to RBD. **h**–**j** The structures of aRBD-2 (**h**), aRBD-5 (**i**) and aRBD-7 (**j**) bound to RBD are also superimposed on that of the RBD:ACE2 complex (PDB ID: 6M0J). The pink circles indicate the clashes between the Nbs and ACE2 (yellow).

To investigate the mechanism by which aRBD-2, aRBD-5, and aRBD-7 neutralize WT SARS-CoV-2, we determined the crystal structures of aRBD-2-7 in complex with WT SARS-CoV-2 RBD-tr2 (tandem repeat RBD-dimer) and aRBD-5 in complex with monomeric WT SARS-CoV-2 RBD at 3.2 Å and 1.8 Å, respectively (Supplementary information, Table S1). According to the structures, aRBD-2 recognizes an epitope close to the lateral loop of the receptor-binding motif (RBM) (Supplementary information, Fig. S1a), which partially overlaps with the epitope of the angiotensin converting enzyme 2 (ACE2) (Fig. 1a). The binding of aRBD-2 with RBD buries a 639.1 Å^2^ surface area. Residues D420, Y421, F456, R457, N460, Y473, Q474, A475, N487, and Y489 of RBD participate in the interaction with aRBD-2 (Fig. 1b). aRBD-5 and aRBD-7 bind to overlapping epitopes on the concave surface anchored by the β-hairpin of the RBM (Supplementary information, Fig. S1b, c), and bury 697.9 and 669.3 Å^2^ surface area, respectively. The epitopes of aRBD-5 and aRBD-7 also partially overlap with that of ACE2 (Fig. 1c, e). Ten (L452, F456, E484, F486, N487, Y489, F490, L492, Q493, and S494) and six (Y449, G482, E484, F490, Q493, and S494) residues of RBD are involved in the interaction with aRBD-5 and aRBD-7, respectively (Fig. 1d, f). The detailed interactions between the Nbs and RBD are shown in Supplementary information, Table S2. Structural superposition indicates that aRBD-2 would not clash with aRBD-5 (Fig. 1g), despite their sharing F456, N487 and Y489 of RBD as contact residues (Supplementary information, Fig. S2). aRBD-2 would not clash with aRBD-7 either, while aRBD-5 and aRBD-7 compete with each other (Fig. 1g). Structural superposition supports that the three Nbs would sterically interfere with the binding of ACE2 to RBD (Fig. 1h–j).

**Fig. 2:**
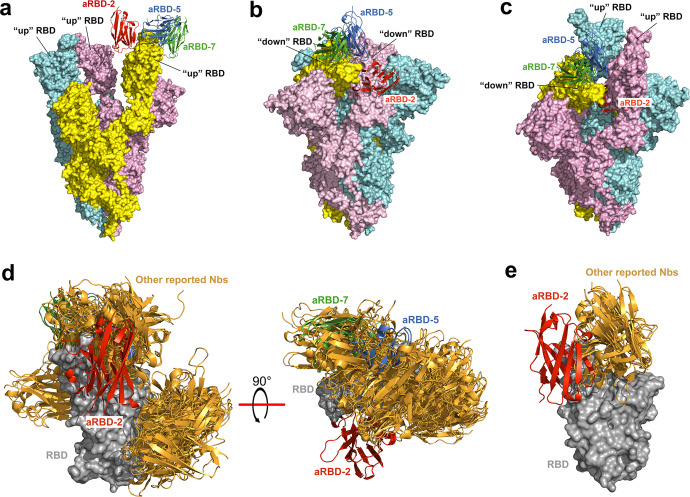
Superposition analysis of the structures of RBD:Nb complexes. The structures of RBD:aRBD-2, RBD:aRBD-5 and RBD:aRBD-7 complex are superimposed on the RBD in the cryo-EM structures of the trimeric spike with all RBD in “up” conformation (**a**, PDB ID: 7KMS), all in “down” conformation (**b**, PDB ID: 7DF3) or two in “up” and one in “down” conformation (**c**, PDB ID: 7KMZ). **d** Alignment of the structures of RBD:aRBD-2, RBD:aRBD-5 and RBD:aRBD-7 complex with other reported RBD/spike:Nb complex structures (bright-orange) deposited in the PDB database. The PDB ID of the other reported RBD/spike:Nb complex structures: 6ZH9, 6ZXN, 7A25, 7B27, 7C8V, 7D2Z, 7D30, 7KGK, 7KKL, 7KLW, 7KN5, 7KN6, 7KN7, 7LX5, 7MEJ, 7MFU, 7MY2, 7MY3, 7N9C, 7N9E, 7N9T, 7NKT, 7OAO, 7OAP, 7OAY, 7OLZ, 7VNB, 7RXD, 7FG3 and 7NLL. **e** Alignment of aRBD-2 with those reported Nbs that clash with aRBD-2 if bound to RBD simultaneously.

To understand the binding mechanism of our Nbs to the intact trimeric spike protein of SARS-CoV-2, we superposed our RBD:Nb complex structures with spike structures in different conformations resolved by cryo-electron microscopy (cryo-EM). All the three Nbs can bind to the “up” RBD in the open conformation of the spike protein (Fig. 2a), while aRBD-5 and aRBD-7 could also bind to the “down” RBD in the inactive conformation of the spike protein, regardless of whether the adjacent RBD is “up” or “down” (Fig. 2b, c). Due to the steric clashes with the adjacent RBD, aRBD-2 cannot bind to the “down” RBD **(**Fig. 2b, c). According to a previously defined classification of SARS-CoV-2 neutralizing antibodies,^48^ aRBD-2 may be classified as a class 1 antibody, while aRBD-5 and aRBD-7 should be class 2 antibodies.

We then use superimposition analysis to study the possible binding mode of the two hetero-bivalent Nbs to the trimeric spike protein. aRBD-2-5 and aRBD-2-7 were constructed by using a flexible linker composed of three repeats of GGGGS (3G_4_S) to fuse aRBD-2 with aRBD-5 or aRBD-7 in a “tail to head” format. The length of the 3G_4_S linker in an extended form is about 54 Å. The observed distance between the C-terminus of aRBD-2 and the N-terminus of aRBD-5 on one single “up” RBD (26.2 Å, Supplementary information, Fig. S3a) or two adjacent “up” RBDs (42.4 Å, Supplementary information, Fig. S3b) is less than the length of the 3G_4_S linker, thus aRBD-2 and aRBD-5 in aRBD-2-5 can simultaneously bind to the same “up” RBD or two different “up” RBDs in the spike. However, aRBD-2 and aRBD-7 in aRBD-2-7 can neither simultaneously bind to one single RBD (the distance is 64.3 Å, Supplementary information, Fig. S3c), nor to two adjacent “up” RBDs (the distance is 80.8 Å, Supplementary information, Fig. S3d). The only binding mode that allows simultaneous binding of the spike by aRBD-2 and aRBD-7 in aRBD-2-7 is that aRBD-2 binds to an “up” RBD, while aRBD-7 binds to the adjacent “down” RBD (the distance is 47.4 Å, Supplementary information, Fig. S3e), which would lock the “down” RBD in the closed state.

aRBD-5 and aRBD-7 share common epitopes and clash with over 50% of the previously described Nbs (PDB ID: 7KGK, 6ZXN, 7D30, 7KN5, 7LX5, 7MEJ, 7OAO, 7OLZ, 7C8V, 6ZH9, 7KM5, 7RXD, 7A25, 7B27, 7JVB, 7KKL, 7KLW, 7MFU, 7OAP, and 7VNB) (Fig. 2d; Supplementary information, Fig. S4), while aRBD-2 binds a unique epitope and only partially clashes with several reported Nbs, including Wnb10 (PDB ID: 7LX5), Re5D06 (PDB ID: 7OLZ), mNb6 (PDB ID: 7KKL), E (PDB ID: 7KN5) and Sb14 (PDB ID: 7MFU) (Fig. 2d, e; Supplementary information, Fig. S4). We also observed that residues mutated in the Omicron variant were extensively bound by each of these previously described Nbs (Supplementary information, Fig. S4). The epitope of aRBD-2 is partially covered by a number of conventional antibodies, including CC12.1 (PDB ID: 6XC2), STE90-C11 (PDB ID: 7B3O), BD-604 (PDB ID: 7CH4), BD-629 (PDB ID: 7CH5), 2B11 (PDB ID:7E5Y), BD-515 (PDB ID: 7E88), C1A-C2 (PDB ID: 7KFX), LY-CoV488 (PDB ID: 7KMH), BG4-25 (PDB ID: 7M6D), ab1 (PDB ID: 7MJJ), PDI 37 (PDB ID: 7MZF), and BETA-27 (PDB ID: 7PS1) (Supplementary information, Fig. S5a). However, aRBD-2 is unique in that all of its binding residues in RBD are conserved in the Omicron variant, while for those conventional antibodies, there are at least two binding residues mutated in the Omicron variant (Supplementary information, Fig. S5b), explaining why except for the BD-629 and BD-515 that have not yet been validated, the other nine of them have been experimentally confirmed to be ineffective or have greatly reduced neutralizing activities against the Omicron variant.^15,18,49–51^

To date (May 31, 2022), the RBD residues that contact aRBD-2 closely are constant in all of the currently or previously circulating SARS-CoV-2 VOCs, variants of interest (VOIs) and variants under monitoring (VUMs), including Alpha (B.1.1.7), Beta (B.1.351, B.1.351 + P384L), Gamma (P.1), Delta (B.1.617.2), Omicron (BA.1, BA.2, BA.3, BA.4, BA.5, XE, and XD), Epsilon (B.1.427), Eta (B.1.525), Theta (P.3), Kappa (B.1.617.1), Lota (B.1.526), Zeta (P.2), Mu (B.1.621), Lambda (C.37), B.1.640, AV.1, AT.1, R.1, B.1.466.2, B.1.1.519, C.36.3, B.1.214.2, B.1.1.523, B.1.616, B.1.619, B.1.620, B.1.630, B.1.1.318 and C.1.2 variants (https://www.who.int/activities/tracking-SARS-CoV-2-variants) (Supplementary information, Fig. S4), suggesting that the epitope of aRBD-2 is highly conserved. To further determine the conservation degree of the aRBD-2 targeting epitope, we analyzed the mutation frequency of these RBD contact residues in the deposited spike proteins from GISAID (Global Initiative of Sharing All Influenza Data) EpiCoV database (September 1, 2021 to February 28, 2022). We found that these epitope residues are highly conserved, with conservation percentage ranging from 99.96639% to 99.99926%, while the conservation percentage of residues E484 and N501 served as analysis controls are only 63.01466% and 63.29653%, respectively (Table 1; Supplementary information, Table S3). These results support that aRBD-2 targets a highly conserved epitope on RBD.

**Table 1:** The conservation of the RBD residues in contact with aRBD-2.

*Defined as the percentage of sequences that do not contain any individual mutation in the 5,971,331 high-quality spike sequences deposited in GISAID (September 1, 2021 to February 28, 2022). Amino acids in red are residues in contact with aRBD-2, and amino acids in blue are used as analysis controls.

### aRBD-2-5 and aRBD-2-7 bind to the RBDs of SARS-CoV-2 variants with high affinities

Mutations that accumulate in RBD render neutralizing antibodies ineffective mainly by eliminating their affinity for the RBD. To explore the impact of the RBD mutations on the bindings of our Nbs, the affinities of aRBD-2, aRBD-5, and aRBD-2-5 for the RBDs of WT SARS-CoV-2 and several major variants, including Alpha, Beta, Gamma, Delta, Delta plus, Kappa, Lambda, Omicron sub-lineages BA.1, and BA.2, were measured by Surface Plasmon Resonance (SPR). The Nb-Fc fusions (fusion of Nbs with human IgG1 Fc) were immobilized on the chip, and kinetics of the RBD flowed over at different concentrations were monitored. Consistent with the structural analysis, aRBD-2-Fc retained the binding to all tested RBDs, with the equilibrium dissociation constant (*K*_D_) ranging from 7.96 to 1.20 nM (Table 2; Supplementary information, Fig. S6). The slightly weaker affinities of aRBD-2-Fc for the RBDs of Beta (*K*_D_ of 3.28 nM), Gamma (*K*_D_ of 4.18 nM) and Delta Plus (*K*_D_ of 2.88 nM) than for WT RBD (*K*_D_ of 1.47 nM) may be caused by the mutation at position K417 that located on the edge of the aRBD-2 binding epitope (Supplementary information, Fig. S7a, b). Besides K417N mutation, S477N mutation presents in the BA.1 and BA.2 is also located on the edge of the aRBD-2-binding epitope. About 5-fold reduced affinities of aRBD-2-Fc for the RBDs of BA.1 and BA.2 may arise from both mutations (Supplementary information, Fig. S7a, b).

**Table 2:** Binding affinity *K*_D_ values (nM) of Nb-Fc proteins for the tested RBDs of SARS-CoV-2 variants detected by SPR.

SARS-CoV-2	aRBD-2-Fc	aRBD-5-Fc	aRBD-2-5-Fc
WT	1.47	2.30	0.0167
Alpha	1.20	3.21	0.0168
Beta	3.28	NB*	0.714
Gamma	4.18	NB	0.668
Delta	1.20	1.90	0.00537
Delta plus	2.88	2.15	<0.001
Kappa	1.40	NB	0.808
Lambda	1.88	NB	0.053
Omicron BA.1	7.96	NB	0.171
Omicron BA.2	7.44	NB	0.0591

^*^NB No binding.

aRBD-5-Fc bound to the RBDs of WT, Alpha, Delta and Delta plus with similar affinities (*K*_D_ ranging from 3.21 to 1.9 nM), but it lost binding to the RBDs of Beta, Gamma, Kappa, Lambda, BA.1 and BA.2 (Table 2; Supplementary information, Fig. S6). Structurally, E484 lies at the center of the aRBD-5 binding epitope, and its mutation may deprive aRBD-5 of binding affinity for the Beta, Gamma and Kappa RBD (Supplementary information, Fig. S7c, d). Besides E484A mutation, BA.1 and BA.2 also encode Q493R mutation, together causing a loss of binding by aRBD-5 (Supplementary information, Fig. S7c, d). aRBD-5 failed to bind to the Lambda RBD, possibly due to the loss of hydrophobic interactions with L452 and F490 (Supplementary information, Fig. S7c, d).

Both aRBD-2-Fc and aRBD-5-Fc retain the binding abilities to the RBDs of WT, Alpha, Delta, and Delta plus. As expected, aRBD-2-5-Fc showed higher binding affinities for these RBDs as compared to the individual aRBD-2-Fc or aRBD-5-Fc, with *K*_D_ ranging from 0.0168 to less than 0.001 nM (Table 2; Supplementary information, Fig. S6), suggesting synergistic effects of the two Nb components. Interestingly, although aRBD-5-Fc lost observable binding to the RBDs of Beta, Gamma, Kappa, Lambda, BA.1 and BA.2, aRBD-2-5-Fc exhibited higher binding affinities for these RBDs than aRBD-2-Fc alone, but by a less extent than those for the RBDs of WT, Alpha, Delta, and Delta plus, with *K*_D_ ranging from 0.808 to 0.053 nM (Table 2; Supplementary information, Fig. S6). A possible explanation for these results is that the fusion with aRBD-2 may bring aRBD-5 close to the RBD to bind non-mutated residues. For example, aRBD-5 loses four hydrogen bond interactions with the RBDs of BA.1 and BA.2 due to E484A and Q493R mutations, but when aRBD-2 pulls aRBD-5 close enough to the Omicron RBDs, there may form one hydrogen bond between Y32 of aRBD-5 and N487 of the RBD, and two hydrogen bonds between S54 of aRBD-5 and S494 of the RBD. Besides, several hydrophobic interactions may also form between Y31 of aRBD-5 with F456 and Y489 of the RBD, between V2, W112 and F115 of aRBD-5 with F486 of the RBD, and between V103, V104 and A105 of aRBD-5 with L452, F490 and L492 of the RBD. These interactions would increase the overall affinities of aRBD-2-5 for the Omicron RBDs (Supplementary information, Fig. S7g). To test this hypothesis, we constructed a fusion protein, aRBD-2-amL1-Fc, by replacing aRBD-5 in aRBD-2-5-Fc with an irrelevant anti-mouse PD-L1 Nb (here named amL1). The results showed that such a fusion failed to increase the binding affinity for the RBD compared to aRBD-2-Fc alone (Supplementary information, Fig. S8).

Since aRBD-7-Fc failed to tolerate the acidic or alkaline solutions used to regenerate SPR chips, enzyme-linked immunosorbent assay (ELISA) was performed to measure the RBD-binding activity of aRBD-7-Fc and aRBD-2-7-Fc, along with aRBD-2-Fc. Consistent with the structural information and SPR results, aRBD-2-Fc bound to all the tested RBDs with 50% maximal effective concentration (EC_50_) ranging from 4.731 to 1.000 nM (Table 3; Supplementary information, Fig. S9a). aRBD-7-Fc tightly bound to the RBDs of WT and Alpha (with EC_50_ of 0.117 nM and 0.141 nM, respectively), but lost the binding to the RBDs of Beta, Gamma, Kappa, Lambda, BA.1 and BA.2, and only weakly bound to the RBDs of Delta and Delta plus (Table 3; Supplementary information, Fig. S9b). These results are well consistent with the structural information. E484 of the RBD is recognized by aRBD-7, and the mutation of this site in Beta, Gamma, Kappa, BA.1 and BA.2 renders aRBD-7 unable to bind to these variant RBDs (Supplementary information, Fig. S7e, f). The L452R mutation in the Delta and Delta plus RBD at the edge of the aRBD-7-binding epitope possibly pushes aRBD-7 away and prevent the binding, while the additional F490S mutation in the Lambda RBD causes aRBD-7 to lose the binding (Supplementary information, Fig. S7e, f). Like aRBD-2-5-Fc, aRBD-2-7-Fc showed increased binding activities to the RBDs that were not or weakly bound by aRBD-7-Fc compared to aRBD-2-Fc (Table 3; Supplementary information, Fig. S9c).

**Table 3:** EC_50_ values (nM) of Nb-Fc proteins binding to the tested RBDs of SARS-CoV-2 variants detected by ELISA.

SARS-CoV-2 RBD	aRBD-2-Fc	aRBD-7-Fc	aRBD-2-7-Fc
WT	1.146	0.117	0.156
Alpha	1.204	0.141	0.191
Beta	4.490	NB*	0.517
Gamma	1.834	NB	0.135
Delta	1.537	ND^#^	0.149
Delta plus	1.330	ND	0.116
Kappa	1.000	NB	0.093
Lambda	1.953	NB	0.165
Omicron BA.1	3.238	NB	0.179
Omicron BA.2	4.731	NB	0.250

^*^NB No binding.

^#^*ND* Not defined.

Taken together, aRBD-2 retains the binding activities to all tested RBDs of major SARS-CoV-2 variants, and its hetero-bivalent constructs by fusing with aRBD-5 or aRBD-7 displayed increased affinities, even though aRBD-5 or aRBD-7 alone is partially ineffective.

### aRBD-2-5-Fc and aRBD-2-7-Fc exhibit potent neutralization activity against SARS-CoV-2 VOCs

The observed high binding affinities prompted us to evaluate the neutralization properties of aRBD-2-5-Fc and aRBD-2-7-Fc against the major variants. Micro-neutralization assay was performed to test the neutralizing activities of aRBD-2-5-Fc and aRBD-2-7-Fc against the authentic Alpha, Gamma and Kappa variants. A previously described Nb with potent neutralization of WT SARS-CoV-2, named Nb21,^39^ was used as a control. Nb21-Fc neutralized Alpha with a 50% inhibitory concentration (IC_50_) of 0.0943 nM, but was inactive against Gamma and Kappa that encode the mutation in E484 (Supplementary information, Fig. S10a–d), which is a binding site for Nb21 (Supplementary information, Fig. S4). aRBD-2-5-Fc and aRBD-2-7-Fc effectively neutralized all of the three variants. Specifically, aRBD-2-5-Fc neutralized Alpha, Gamma and Kappa with IC_50_ of 0.0511, 0.1087, and 0.0769 nM, respectively, while aRBD-2-7-Fc with IC_50_ of 0.0328, 0.0045 and 0.1914 nM, respectively (Supplementary information, Fig. S10a–d). Compared to Nb21-Fc, aRBD-2-5-Fc and aRBD-2-7-Fc showed 1.8- and 2.9-fold higher neutralizing activity against Alpha, respectively.

**Fig. 3:**
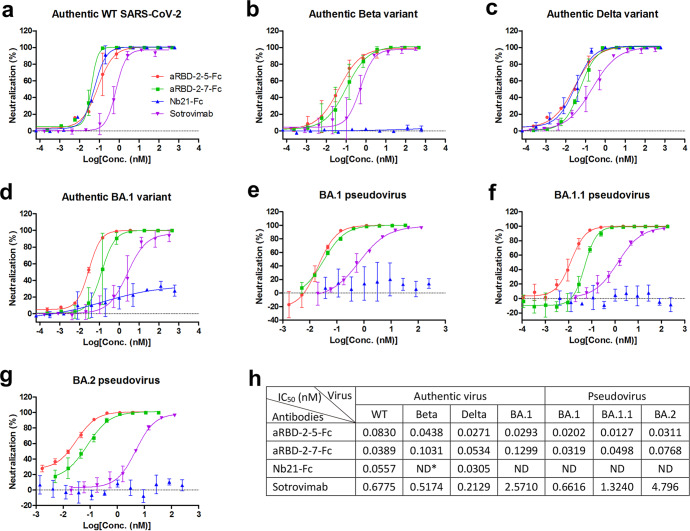
Evaluation of neutralization properties of aRBD-2-5-Fc and aRBD-2-7-Fc against authentic or pesudotyped SARS-CoV-2. **a**–**g** Authentic WT SARS-CoV-2 (**a**), Beta (**b**), Delta (**c**), and Omicron BA.1 (**d**) and pseudotyped Omicron BA.1 (**e**), BA.1.1 (**f**) and BA.2 (**g**) were neutralized with serially diluted antibodies. Error bars indicate the means ± SD from two (authentic virus) or three (pseudovirus) independent experiments. **h** IC_50_ values were calculated by fitting the neutralization (%) values of serial dilution with a sigmoidal dose-response curve. *ND, neutralization not detected.

Plaque reduction neutralization test (PRNT) was performed to assess the neutralization properties of aRBD-2-5-Fc and aRBD-2-7-Fc against the authentic WT, Beta, Delta and Omicron BA.1 variants (Fig. 3a–d, h). In addition to Nb21-Fc, a conventional antibody Sotrovimab (S309^52^), authorized for emergency use by the U.S. Food and Drug Administration (FDA), was also used as a control. Nb21-Fc neutralized WT virus and Delta with IC_50_ of 0.0557 and 0.0305 nM, respectively, but failed to neutralize Beta and BA.1 (Fig. 3a–d, h). The neutralizing activities of aRBD-2-5-Fc and aRBD-2-7-Fc against WT virus and Delta were comparable to those of Nb21-Fc (Fig. 3a, c, h). Specifically, compared to Nb21-Fc, aRBD-2-5-Fc and aRBD-2-7-Fc showed 1.5-fold lower (IC_50_ of 0.0830 nM) and 1.4-fold higher (IC_50_ of 0.0389 nM) activity in neutralizing WT virus, and 2.0-fold higher (IC_50_ of 0.0271 nM) and 1.8-fold lower (IC_50_ of 0.0534 nM) activity in neutralizing Delta, respectively (Fig. 3h). aRBD-2-5-Fc also exhibited strong neutralization against BA.1 with an IC_50_ of 0.0293 nM, even 2.8-fold more potency than its activity against WT virus (Fig. 3a, d, h). Compared with aRBD-2-5-Fc, aRBD-2-7-Fc was 4.4-fold less potent in neutralizing BA.1, with an IC_50_ of 0.1299 nM (Fig. 3d, h). Both aRBD-2-5-Fc and aRBD-2-7-Fc showed much higher activities than Sotrovimab in neutralizing the tested viruses (Fig. 3a–d, h). Especially for neutralizing BA.1, aRBD-2-5-Fc and aRBD-2-7-Fc exhibited 88- and 20-fold higher potency than Sotrovimab, respectively (Fig. 3d, h).

Considering that Omicron BA.2 has replaced BA.1 as the main circulating virus from 2022^53^ (as of May 31, 2022), we further tested our antibodies for neutralizing against the BA.2 variant, along with the BA.1 and another sub-lineage BA.1.1, by using HIV-1-based pseudoviruses (Fig. 3e–h). Similar to the results with the authentic virus, Nb21-Fc showed no neutralization against the three pseudotyped viruses (Fig. 3e–h). aRBD-2-5-Fc neutralized the three viruses the best with comparable potency (IC_50_ of 0.0202, 0.0127 and 0.0311 nM against BA.1, BA.1.1 and BA.2, respectively). aRBD-2-7-Fc was 1.6- to 3.9-fold less potent than aRBD-2-5-Fc (IC_50_ of 0.0319, 0.0498, and 0.0768 nM against BA.1, BA.1.1 and BA.2, respectively) (Fig. 3e–h). Sotrovimab retained the neutralizing activities against the three viruses, but its neutralizing potency against BA.2 was 7.2-fold lower than against BA.1 (Fig. 3e–h). Consistent with the results with the authentic virus, aRBD-2-5-Fc and aRBD-2-7-Fc showed much higher activities than Sotrovimab. Specifically, aRBD-2-5-Fc was 33-, 104-, and 154-fold more potent, and aRBD-2-7-Fc was 21-, 27-, and 62-fold more potent than Sotrovimab in neutralizing BA.1, BA.1.1, and BA.2, respectively (Fig. 3h).

Taken together, these cellular assays demonstrated that our hetero-bivalent Nbs retain strong neutralization activity against all the tested major variants, including Alpha, Beta, Gamma, Kappa, Delta, Omicron BA.1, BA.1.1 and BA.2.

### aRBD-2-5-Fc provides preventive protection against WT and mouse-adapted SARS-CoV-2 in mice

**Fig. 4:**
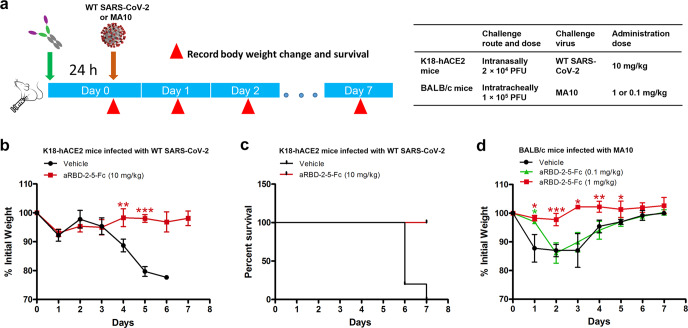
aRBD-2-5-Fc provide prophylactic protection against WT and mouse-adapted SARS-CoV-2 in mice. **a** Animal experiment scheme. **b**, **c** Body weight change (**b**) and survival (**c**) of K18-hACE2 mice treated with aRBD-2-5-Fc at a single dose of 10 mg/kg (*n* = 5) or PBS (*n* = 5) followed by i.n. infection with 2 × 10^4^ PFU of WT SARS-CoV-2. **d** Body weight change of BALB/c mice treated with aRBD-2-5-Fc at doses of 1 mg/kg (*n* = 4), 0.1 mg/kg (*n* = 4) or PBS (*n* = 4) followed by intratracheal infection with 1 × 10^5^ PFU of MA10. Error bars indicate means ± SD. Unpaired *t*-test with a Welch’s correction was used for statistical analysis of significance of the difference between the treated group and the control group, *P  ≤  0.05, **P  ≤  0.01, ***P  ≤  0.001.

As aRBD-2-5-Fc exhibited potent neutralization of SARS-CoV-2 in vitro, we then sought to test it for protection in vivo. Firstly, we tested aRBD-2-5-Fc for prophylactic protection against WT SARS-CoV-2 in K18-hACE2 mice (which is a severe disease model) expressing human ACE2 under the cytokeratin-18 promoter.^54^ The animals were intraperitoneally (i.p.) injected with single dose of 10 mg per kg body weight (hereafter, mg/kg) of aRBD-2-5-Fc or phosphate buffer saline (PBS, vehicle control). 24 h later, the animals were intranasally (i.n.) inoculated with 2 × 10^4^ PFU of WT SARS-CoV-2 and monitored for 7 days (Fig. 4a). The control animals treated with PBS showed significant body weight loss from day 4 post infection, and most animals lost approximately 20% of their body weight by 5 days post infection (dpi) (Fig. 4b). Severe mortalities caused by the infection occurred in the control animals: 80% of them were dead by 6 dpi, and the mortality reached 100% at 7 dpi (Fig. 4c). In contrast, neither significant body weight loss nor mortality was observed in the animals treated with aRBD-2-5-Fc (Fig. 4b, c).

We next tested a lower dose of aRBD-2-5-Fc for prophylactic protection against mouse-adapted SARS-CoV-2 virus MA10 in BALB/c mice. MA10 encodes Q493K, Q498Y and P499T mutations in the RBD, among which, Q493 is located in the binding epitope of aRBD-5. Unlike the high mortality rate of the K18-hACE2 mouse model, MA10 infection only resulted in a mortality rate of ~15% in young BALB/c mice, despite acute lung injury and significant weight loss.^55^ Animals were i.p. administered with single dose of 1 or 0.1 mg/kg of aRBD-2-5-Fc followed by intratracheal inoculation with 1 × 10^5^ PFU of the MA10. Animals mock-treated with PBS were set as controls (Fig. 4a). As shown in Fig. 4d, the control animals rapidly lost on average of 12.2% of initial body weight at 1 dpi, and reached maximum body weight loss of 13.0% at 2 dpi and maintained the loss of 13.0% at 3 dpi. Animals treated with 0.1 mg/kg of aRBD-2-5-Fc lost only on average of 3.0% of initial body weight at 1 dpi, but reached maximum body weight loss of 13.9% at 2 dpi. In contrast, only an average of 1.7% of initial body weight loss at 1 dpi and maximum body weight loss of 2.2% at 2 dpi was observed in the animals treated with 1 mg/kg of aRBD-2-5-Fc. All the animals survived to the end of the study. Taken together, these results demonstrate that aRBD-2-5-Fc provides prophylactic protection against SARS-CoV-2 in mice, even administered one dose as low as 1 mg/kg.

### aRBD-2-5-Fc eliminates infectious Omicron BA.1 virus in hamsters

**Fig. 5:**
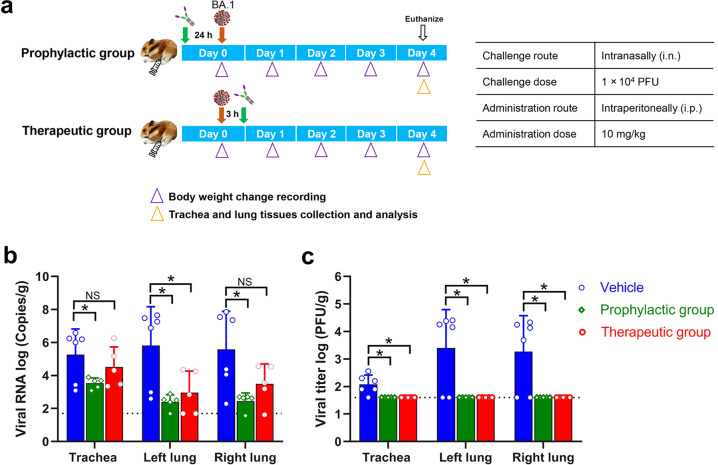
aRBD-2-5-Fc offers prophylactic and therapeutic protection against Omicron BA.1 in hamsters. **a** Animal experiment scheme. Hamsters were administered (i.p.) with aRBD-2-5-Fc at 10 mg/kg 24 h before (prophylactic group, *n* = 5) or 3 h after (therapeutic group, *n* = 5) intranasal inoculation with 1 × 10^4^ PFU of Omicron BA.1 virus. Hamsters administered with PBS (*n* = 6) were set as controls. **b**, **c** Viral RNA (**b**) and infectious virus titers (**c**) in the trachea and lungs were quantified at 4 dpi. Dashed lines indicate the detection limit of the assay. Error bars indicate means ± SD. Unpaired *t*-test with a Welch’s correction was used for statistical analysis of significance of the difference between the treated group and the control group. NS, no significant; *P ≤ 0.05.

The Omicron variant is currently circulating globally, so we further tested aRBD-2-5-Fc for protection against this variant using a Syrian golden hamster model. aRBD-2-5-Fc was i.p. administered at 10 mg/kg to hamsters 24 h before (prophylactic group) or 3 h after (therapeutic group) i.n. inoculation with 1 × 10^4^ PFU of Omicron BA.1 virus. The animals treated with PBS were set as controls (Fig. 5a). Viral RNA copies (Fig. 5b) and infectious virus titers (Fig. 5c) in the trachea and lungs of the animals were determined at 4 dpi. Compared with the control group, prophylactic-treated animals had significantly fewer viral RNAs in trachea, left lung and right lung by 10^1.73^-, 10^3.41^- and 10^3.13^-fold, respectively, while therapeutic group had less reduction, reduced by 10^0.75^-, 10^2.84^- and 10^2.09^-fold, respectively (Fig. 5b). Importantly, no infectious virus was detected in both treatment groups, but an average of 10^2.08^, 10^3.40^ and 10^3.27^ PFU/g of infectious viruses were still present in the control animals’ trachea, left lung and right lung tissues, respectively (Fig. 5c). No body weight loss after infection was observed (Supplementary information, Fig. S11), as the Omicron BA.1 variant causes only attenuated disease in hamsters.^56^

### aRBD-2-5-Fc is pharmacokinetically stable in mice and hamsters

Given that aRBD-2-5-Fc offered effective protection in mice and hamsters against SARS-CoV-2, its pharmacokinetic profiles were further determined in these two animal models. A single-dose of aRBD-2-5-Fc (10 mg/kg) was i.p. administered to the animals, and plasma antibody concentrations at various time points after the administration were quantified by ELISA. The pattern of aRBD-2-5-Fc concentrations in the plasma of both mice and hamsters showed linear pharmacokinetic characteristics, and the estimated half-life of aRBD-2-5-Fc is 165 h in mice and 72 h in hamsters, indicating that aRBD-2-5-Fc is very stable in vivo (Supplementary information, Fig. S12a). All animals survived to the end of the study with no significant body weight loss (Supplementary information, Fig. S12b).

## Discussion

SARS-CoV-2 continues to evolve, and multiple sub-lineages of the Omicron variant, including BA.1, BA.2, BA.3, BA.4, BA.5 and their derivatives, have been identified around the world (as of May 31, 2022). These Omicron sub-lineages have accumulated mutations at 21 sites in the RBD of the spike protein, including G339, R346, S371, S373, S375, T376, D405, R408, K417, N440, G446, L452, S477, T478, E484, F486, Q493, G496, Q498, N501, and Y505 (https://www.who.int/activities/tracking-SARS-CoV-2-variants; https://www.ncbi.nlm.nih.gov/activ), which results in evasion of neutralizing antibodies. Antibodies targeting conserved RBD epitopes and rational design strategies to enhance the breadth and activity of neutralizing antibodies are needed to help fight the epidemic.

X-ray crystallography structures revealed that aRBD-2 binds to an RBM epitope consisting of ten contact residues, including D420, Y421, F456, R457, N460, Y473, Q474, A475, N487, and Y489 (Fig. 1b; Supplementary information, Table S2). These residues are highly conserved in currently or previously circulating VOCs, VOIs and VUMs (Supplementary information, Fig. S4), and their high conservation is further confirmed with bioinformatic analysis on almost 6 million sequences from GISAID EpiCoV database (Table 1). Deep mutational scanning on individual residues of SARS-CoV-2 RBD conducted by Starr et al.^57^ revealed that any substitution at D420, F456, R457, Q474, N487 or Y489 would reduce RBD expression and/or its affinity for ACE2, and that only Y421F, Y473F and A475G mutations present individually did not impair the RBD functions, providing an additional support that these residues are highly conserved. The only exception is N460, which tolerates substitution with P, V, I, A, T, S, H, K, or R, consistent with our analysis that N460 is least conserved in the 10 residues, with the conservation rate of 99.96639% in the GISAID database (Table 1).

Antibody cocktail consisting of neutralizing antibodies that bind distinct epitopes is a potentially effective strategy against viral escape,^58,59^ and used to be the main clinical countermeasures against COVID-19, e.g., Casirivimab/Imdevimab, Cilgavimab/Tixagevimab, Bamlanivimab/Etesevimab, and Amubarvimab/Romlusevimab.^60–62^ Unfortunately, both component antibodies of these cocktails were escaped by the Omicron variant, resulting in a dramatic reduction in overall activity.^17,49^ Another effective strategy against variant escape is multivalent antibodies engineered through connecting antibodies targeting distinct epitopes together. Multivalent antibodies have potential advantages over antibody cocktails. Firstly, as single molecules, multivalent antibodies have cost advantage due to simpler formulation and manufacture.^63^ Secondly, multivalent antibodies have greatly enhanced overall binding affinity as different antibody components can bind more epitope sites simultaneously, thereby being more resistant to escaping variants. Our hetero-bivalent antibody aRBD-2-5-Fc bound the WT RBD with an affinity *K*_D_ of 0.0167 nM, which is 88- to 138-fold higher than its component aRBD-2-Fc and aRBD-5-Fc, respectively (Table 2). Importantly, even though aRBD-5 itself lost apparent binding to some of the variants, the overall affinities of aRBD-2-5-Fc for these variants were higher than those of aRBD-2-Fc alone, indicating that the remaining non-mutated epitope residues in these variants can still bind aRBD-5 and contribute to the overall affinity (Table 2). Attaining high affinity and more ACE2-competition from the synergistic effect of the component Nbs, aRBD-2-5-Fc potently neutralizes all the VOCs (Fig. 3; Supplementary information, Fig. S10).

In addition to the advantages of lower production cost, higher stability, and easier genetic manipulation, Nbs are suitable candidates for therapeutic applications and valuable alternative to conventional antibodies.^46^ In February 2019, the U.S. FDA approved a bivalent Nb, Caplacizumab, for the treatment of thrombotic thrombocytopenic purpura and thrombosis. A panel of Nbs prepared in different forms such as Fc labeling and bispecificity are being confirmed in clinical trials.^64^ Since camelid VHH has high degree of homology with human VH_3_, Nbs generally have low immunogenicity and thus are suitable for human administration. Nonetheless, the sequences of therapeutic Nbs can always be “humanized” if necessary.^65^ The small size (~15 kDa) allows Nbs to minimize steric hindrance issues when made into multivalent antibodies, which makes Nbs more suitable than conventional antibodies for developing multivalent antibodies targeting the few remaining non-mutated epitopes on the RBD of SARS-CoV-2 variants. Several conventional antibodies occupy most of the epitope residues of aRBD-2, but they still failed to combat the challenge from Omicron variant, while aRBD-2 did (Supplementary information, Fig. S5). This inspires us to continue to develop more Nbs and then combine them in cocktail or with multivalent strategies to defeat the epidemic.

In this study, pseudovirus assay showed that aRBD-2-5-Fc is ~154-fold more potent than Sotrovimab in neutralizing the Omicron BA.2, with IC_50_ of 31.1 pM (or 3.45 ng/mL) (Fig. 3h). According to a recent study,^17^ no authorized monoclonal antibody therapy could adequately neutralize Omicron BA.1 and BA.2 except the recently authorized antibody LY-CoV1404. LY-CoV1404 was ~200-fold more potent than Sotrovimab in neutralizing the pseudotyped Omicron BA.2, with an IC_50_ of ~5 ng/mL, indicating that aRBD-2-5-Fc and LY-CoV-1404 may have comparable neutralizing potency against this currently major circulating variant, BA.2. PRNT assay results showed that aRBD-2-5-Fc neutralized 75 PFU of authentic Omicron BA.1 with IC_50_ of 29.3 pM (or 3.23 ng/mL) (Fig. 3h), while PRNT assay conducted by Westendorf K et al.^66^ showed that LY-CoV1404 neutralized 75 PFU of authentic Omicron BA.1 with IC_50_ of 16 ng/mL (or 111.1 pM), indicating that aRBD-2-5-Fc may be slightly more potent than LY-CoV1404 in neutralizing BA.1. Besides LY-COV1404, conventional antibodies XGv289 and XGv347 reported by Wang K et al.^67^ and 87G7 reported by Du W et al.^68^ also showed potent neutralization against Omicron BA.1 and/or BA.2. Structural superposition shows that the RBD-binding epitopes of aRBD-2 and aRBD-5 do not overlap with those of LY-COV1404 and XGv289, but partially overlap with those of XGv347 and 87G7 (Supplementary information, Fig. S13). Another recent study^69^ reported a bispecific Nb bn03 with broad neutralization against the WT SARS-CoV-2 and its VOCs. Possibly due to non-competition with ACE2 of the both components, bn03 showed moderate neutralizing potencies, with IC_50_ ranging from 0.11 to 0.76 μg/mL. However, bn03 delivered via inhalation exhibited effective protection against SARS-CoV-2 in mouse models, which highlights the clinical potential of bispecific Nbs via inhalation administration. This delivery route of bispecific Nbs should also be tested for our aRBD-2-5 in the future.

This study has several limitations. Firstly, we found that aRBD-5 lost binding to some of the variants but still contributes to the overall binding when fused to aRBD-2. The precise mechanism will require further determination on the structure of aRBD-2-5 in complex with the variant RBDs. Secondly, we assessed the antiviral effect of aRBD-2-5-Fc on Omicron BA.1 in hamsters and measured the viral titers in trachea and lung tissue at 4 dpi, when the viral load in control animals is relatively low. It may be better to sample the viral loads at an earlier time point. Thirdly, only one dose of 10 mg/kg was tested for prophylactic and therapeutic protection of aRBD-2-5-Fc against Omicron BA.1 in hamsters. The most appropriate dose will need to be determined in the future.

## Materials and methods

### Protein preparation

SARS-CoV-2 RBD (amino acids 321 to 591 of sipke) proteins used for characterizing the binding properties of our Nbs were prepared as our previous study.^47^ Briefly, the coding sequences of WT RBD (YP_009724390.1), Alpha (N501Y), Beta (K417N, E484K, N501Y), Gamma (K417T, E484K, N501Y), Delta (L452R, T478K), Delta plus (K417N, L452R, T478K), Kappa (L452R, E484Q) and Lambda (L452Q, F490S) variants were cloned into pTT5 vector, with the C terminal containing a TEV cleavage site and a human IgG1 Fc. The recombinant vectors were transiently transfected into HEK293F cells with polyethyleneimine (Polyscience). After three days of expression, fusion protein was purified from the cell supernatant using protein A column (GE healthcare). After digestion with TEV protease, the Fc fragment was removed by a second protein A column purification, and the TEV protease was removed by a Nickel column (GE healthcare). WT SARS-CoV-2 RBD (amino acids 321 to 528) and mouse PD-L1 extracellular domain (ADK70950.1, amino acids 19 to 239) proteins were prepared similarly. Nbs, hetero-bivalent Nbs, IgG1 Fc-fused Nbs and IgG1 Fc-fused hetero-bivalent Nbs, including aRBD-2, aRBD-5, aRBD-7, aRBD-2-5, aRBD-2-7, amL1-Fc, aRBD-2-Fc, aRBD-5-Fc, aRBD-7-Fc, aRBD-2-5-Fc, aRBD-2-7-Fc and aRBD-2-amL1-Fc, were also prepared similarly. All recombinant vectors were constructed using Gibson Assembly method.^70^ Omicron BA.1 and BA.2 RBD (amino acids 319 to 541) proteins were purchased from Sino Biological. WT RBD-tr2 (amino acids 319 to 537, tandem repeat dimer) protein was kindly provided by Anhui Zhifei Longcom Biopharmaceutical Co., Ltd.

### Crystallization and data collection

WT SARS-CoV-2 RBD (amino acids 321 to 528) was mixed with aRBD-5 and RBD-tr2 was mixed with aRBD-2-7 in a molar ratio of 1:1.2 to form complexes. To remove excess Nbs, the mixtures were further purified by gel filtration. The complex protein was concentrated to 20 mg/mL for crystallization screening. Sitting-drop vapor diffusion method was applied to obtain the crystals of complexes by mixing 0.2 µL of complex protein with an equal volume of reservoir solution. Optimized crystal of RBD:aRBD-5 complex was achieved in 0.1 M sodium cacodylate at pH 5.5, 25% (w/v) PEG 4000 for about 1 month at 18 °C, while crystal of RBD-tr2:aRBD-2-7 complex was grown in 0.1 M (NH_4_)_2_SO_4_, 0.1 M Tris-HCl, pH 7.5, 20% (w/v) PEG1500 for about 1 month at 18 °C. For data collection, single crystals were flashed-cooled in liquid nitrogen after immersed in the cryoprotectant composed of 15% (v/v) glycerol for the crystals of RBD-tr2:aRBD-2-7 complex, and 20% (v/v) ethylene glycol for the crystals of RBD:aRBD-5 complex in the containing reservoir solution for few seconds. Diffraction data were collected at Shanghai Synchrotron Radiation Facility (SSRF) beamline BL19U1 for RBD:aRBD-5 complex at the wavelength of 0.97852 Å and BL02U1 for RBD-tr2:aRBD-2-7 complex at the wavelength of 0.97918 Å, respectively.

### Structural determination

Data were processed with XDS.^71^ Initial phases were solved by molecular replacement method with Phaser^72^ from the CCP4i program package,^73^ using SARS-CoV-2 RBD/ACE2 (PDB ID: 6M0J) and RSV/F-VHH-4 (PDB ID: 5TP3) as search models for aRBD-5 in complex with RBD, and SARS-CoV-2 RBD/ACE2 (PDB ID: 6M0J), TcdB-B1/B39 VHH (PDB ID:4NC2) and Vsig4/Nb119 (PDB ID: 5IMK) were used as search models for RBD-tr2 complexed with aRBD-2-7. Subsequent model building and refinement were achieved using COOT and Phenix.^74^ The structural data of RBD-tr2:aRBD-2-7 and RBD:aRBD-5 complexes have been deposited in the Protein Data Bank under accession codes 7FH0 and 7VOA. All structural figures were prepared by PyMOL.

### Conservation analysis of the RBD residues interacted with aRBD-2

To estimate the conservation of the RBD residues in contact with aRBD-2, we analyzed data from GISAID EpiCoV database,^75^ focusing on spike protein sequences submitted in a 6-month window from September 2021 to February 2022. We first filtered out low quality sequences with unknown amino acids, and further kept high quality sequences with more than 1200 amino acids. Totally 5,971,331 sequences were kept for further alignments with the RBD of the SARS-CoV-2 reference protein sequence using NCBI Protein BLAST command tool.^76^ Following statistical analyses were performed on the ten RBD residues in contact with aRBD-2 and two control residues (E484 and N501) using custom Shell and R scripts.

### SPR

SPR measurements were performed at 25 °C using a BIAcore T200 system. Nb-Fc was diluted to a concentration of 5 μg/mL with sodium acetate (pH 4.5) and immobilized on a CM5 chip (GE Healthcare). All proteins were exchanged into the running buffer (PBS containing 0.05% Tween-20, pH 7.4), and the flow rate was 30 μL/min. The blank channel of the chip served as the negative control. For affinity measurements, a series of different concentrations of RBD flowed over the sensorchip. After each cycle, the chip was regenerated with 50 mM NaOH buffer for 60 to 120 s. The sensorgrams were fitted with a 1:1 binding model using Biacore evaluation software.

### ELISA

SARS-CoV-2 RBD was coated onto Immuno-MaxiSorb plates (Nunc) at final concentration of 2 μg/mL for 2 h at 4 °C. The plates were washed with PBS then blocked with MPBS (PBS containing 5% defatted milk) for 2 h at room temperature. Serially diluted Nb-Fc solutions were added to the plates, followed by incubation for 1 h at room temperature. After four washes with PBST (PBS containing 0.1% tween-20), bound Nb-Fc was detected with HRP-conjugated anti-human IgG1 Fc antibody (Sino Biological). After incubation for 1 h at room temperature, the plates were washed 4 times with PBST. 100 μL per well TMB (Beyotime) was added and reacted under dark for 5 min, and 50 μL per well of 1 M H_2_SO_4_ was added to stop the reaction. OD_450_ was read by a Synergy H1 plate reader (Biotek). The data was analyzed using GraphPad Prism software.

### Authentic SARS-CoV-2 neutralization assay

A micro-neutralization assay by counting infected cells was employed to evaluate the neutralizing activity of Nb-Fc fusions against authentic Alpha, Gamma or Kappa variants. Briefly, Nb-Fc in a 3-fold dilution concentration series was incubated with 200 PFU of SARS-CoV-2 Alpha (England 204820464/2020), Gamma (Japan TY7-503/2021) and Kappa (USA/CA-Stanford-15_S02/2021) virus for 30 min. The antibody-virus mixtures were then added to Vero E6 cells in 96-well plates (Corning). After 1 h, the supernatant was removed, and the cells were washed with PBS and overlaid with Dulbecco modified Eagle medium (DMEM) containing 0.5% methylcellulose. Cells infected with virus without antibody addition were used as virus controls. After 2 days of infection, the cells were fixed with 4% paraformaldehyde, permeabilized with 0.1% Triton-100, blocked with DMEM containing 10% fetal bovine serum, and stained with a rabbit monoclonal antibody against SARS-CoV-2 NP (GeneTex, GTX635679) and an Alexa Fluor 488-conjugated goat anti-mouse secondary antibody (Thermo Fisher Scientific). Fluorescence images of the entire well were acquired with a 4× objective in a Cytation 5 (BioTek). The infected cells indicated by the NP staining were quantified with the cellular analysis module of the Gen5 software (BioTek). Infection (%) = sample infected cell number/virus control infected cell number (%). The IC_50_ was analyzed using GraphPad Prism software.

Neutralizing activities of Nb-Fc fusions against WT SARS-CoV-2, Beta, Delta and Omicron BA.1 variants were evaluated using PRNT assay as our previous study^77^ with slight modification. Briefly, Vero E6 cells were seeded overnight in 24-well culture plates at 1.5 × 10^5^ per well. Nb-Fc were serially diluted five-fold in DMEM containing 2.5% FBS were incubated with equal volume of 75 PFU of SARS-CoV-2 WT virus (IVCAS 6.7512), Beta virus (NPRC2.062100001), Delta virus (GWHBEBW01000000) or Omicron virus (CCPM-B-V-049-2112-18) at 37 °C for 1 h, respectively. Then, the mixture was added to the cells. Cells infected with virus without antibody addition were used as virus controls. After an additional 1 h incubation at 37 °C, the antibody-virus mixture was removed, and DMEM containing 2.5% FBS and 0.9% carboxymethy lcellulose were added. Plates were fixed with 8% paraformaldehyde and stained with 0.5% crystal violet and rinsed thoroughly with water 3 days later. Plaques were then enumerated, and the neutralization percentage was calculated by the formula: Neutralization (%) = (1 – sample plaques/virus control plaques) (%). The IC_50_ was analyzed using GraphPad Prism software.

### Pseudovirus neutralization assay

Pseudoviruses were used to evaluate the neutralizing activities of Nb-Fc fusions against Omicron BA.1, BA.1.1 and BA.2. HIV-1-based pseudoviruses that carry BA.1.1 spike and luciferase reporter gene was purchased from Sino Biological, and HIV-1-based pseudotyped BA.1 and BA.2 were kindly provided by Professor Wei Chen’s Lab, Beijing Institute of Biotechnology. Pseudovirus neutralization assays were performed as described before.^78^ Briefly, ACE2-293T cells were cultured overnight in 96-well plates at 2.5 × 10^4^ per well. The antibodies threefold serially diluted with DMEM plus 10% FBS were incubated with pseudovirus dilution of relative light unit (RLU) at around 350,000 at 37 °C for 1 h. The antibody-pseudovirus mixtures were then added to the monolayer ACE2-293T cells. After 2-day culture, the cells were lysed and treated using Bright-Lite detection reagent (Vazyme, DD1204). Luciferase activity was measured by a microplate luminescence detector (TECAN, SPARK 10 M). Cells without virus and antibodies were used as blank controls, and cells without antibodies were used as virus controls. The neutralization percentage was calculated by the formula: Neutralization (%) = [1 − (sample RLU − Blank control RLU)/(Virus Control RLU − Blank control RLU)] (%). The IC_50_ was calculated using GraphPad Prism software.

### Mouse study

Male K-18 hACE2-transgenic mice (11–12 weeks of age, from the Jackson Laboratory) were i.p. administered with one dose of aRBD-2-5-Fc (10 mg/kg) or PBS 24 h before i.n. inoculation with 2 × 10^4^ PFU of WT SARS-CoV-2. BALB/c mice (8 weeks of age) of both sexes were i.p. administered with one dose of aRBD-2-5-Fc (1 mg/kg or 0.1 mg/kg) 24 h before intratracheal inoculation with 1 × 10^5^ PFU of mouse-adapted SARS-CoV-2 (MA10). Animals were weighed daily and considered moribund if they lost 20% of their initial body weight as per institutional IACUC regulation. Animals were euthanized by isoflurane overdose or Euthasol (89 mg/kg) injection at the end of the study. All operations were performed in the biosafety level 3 (BSL-3) facility and approved by the Institutional Animal Care and Use Committee at UT-Health San Antonio (assurance number: 2020040AR and 2020048AR). The following reagent was obtained through BEI Resources, NIAID, NIH: SARS-Related Coronavirus 2, Mouse-Adapted MA10 Variant (in isolate USA-WA1/2020 backbone), Infectious Clone (ic2019-nCoV MA10) in Calu-3 Cells, NR-55329, contributed by Ralph S. Baric.

### Hamster study

For prophylactic evaluation, female Syrian golden hamsters (5–6 weeks of age) were i.p. administrated with one dose of 10 mg/kg of aRBD-2-5-Fc (*n* = 5) after anesthetized by chamber induction (5 L 100% O_2_/min and 3%–5% isoflurane). 24 h later, the animals were i.n. infected with 1 × 10^4^ PFU of SARS-CoV-2 Omicron virus in 100 μL of PBS. For therapeutic evaluation of aRBD-2-5-Fc, female Syrian golden hamsters (5–6 weeks of age) were i.n. challenged with 1 × 10^4^ PFU of SARS-CoV-2 Omicron virus. 3 h later, the animals were i.p. administrated with one dose of 10 mg/kg of aRBD-2-5-Fc (*n* = 5). Animals i.p. administrated with PBS were set as control (*n* = 6). Animals were weighed daily and euthanized by isoflurane overdose at 4 dpi, tissues (trachea and lungs) were harvested for analysis of virus RNA copies and titers. All operations were performed in BSL-3 facility, and the protocols were approved by the Institutional Review Board at Wuhan Institute of Virology (assurance number: WIVAF45202202).

### Virus RNA copies and titers

Viral RNA in the tissue homogenates was quantified by one-step real-time RT-PCR as described before.^79^ Briefly, viral RNA was purified using the QIAamp Viral RNA Mini Kit (Qiagen), and quantified with HiScript® II One Step qRT-PCR SYBR® Green Kit (Vazyme Biotech Co., Ltd.) with the primers ORF1ab-F (5ʹ-CCCTGTGGGTTTTACACTTAA-3ʹ) and ORF1ab-R (5ʹ-ACGATTGTGCATCAGCTGA-3ʹ). The amplification procedure was set up as: 50 °C for 3 min, 95 °C for 30 s followed by 40 cycles consisting of 95 °C for 10 s, 60 °C for 30 s.

Virus titer was determined with plaque assay as previously described with slight modification.^77^ Briefly, virus samples were serially ten-fold diluted with DMEM containing 2.5% FBS, and inoculated to Vero E6 cells cultured overnight at 1.5 × 10^5^/well in 24-well plates; after incubating at 37 °C for 1 h, the inoculate was replaced with DMEM containing 2.5% FBS and 0.9% carboxymethyl-cellulose. The plates were fixed with 8% paraformaldehyde and stained with 0.5% crystal violet 3 days later. Virus titer was calculated with the dilution gradient with 10–100 plaques.

### In vivo half-life measurement

The in vivo half-life of aRBD-2-5-Fc was measured in mice and hamsters. Four male C57BL/6 mice (7–8 weeks of age) and three female Syrian golden hamsters (5**–**6 weeks of age) were i.p. injected with aRBD-2-5-Fc at single dose of 10 mg/kg, respectively. Hamster blood was collected from the eye socket venous plexus by a capillary tube at different time points post-injection, while mouse blood was collected from the cut tip of the tail. Animals were weighed daily and euthanized by isoflurane overdose at the end of the study. The antibody concentrations in plasma were detected using ELISA as described above with aRBD-2-5-Fc as standard. Briefly, 1:10,000 dilutions of plasma and 1:2 serial dilutions of aRBD-2-5-Fc (0.03 μg/mL starting concentration) were added to WT SARS-CoV-2 RBD-coated immuno-plates. The bound aRBD-2-5-Fc was detected with HRP-conjugated anti-human IgG1 Fc antibody. Data were analyzed using Microsoft Office Excel. The linear portions of standard curves were used to quantify the aRBD-2-5-Fc in the plasma samples. The aRBD-2-5-Fc concentrations in plasma were plotted as their natural logarithms against time. The resulting curves followed a two-compartment model, and the half-life was calculated from the slopes. The protocols were approved by the Institutional Animal Use and Care Committee of University of Science and Technology of China (assurance number: USTCACUC22220122022).

### Statistical analysis

All statistical analyses were performed in GraphPad Prism 9.0. An unpaired *t*-test with a Welch’s correction for unequal standard deviations was used for comparisons of two groups. The asterisks shown in the figures refer to the level of significance: *P  ≤  0.05; **P  ≤  0.01; ***P  ≤  0.001.

## Supplementary information

Supplementary information, Fig. S1

Supplementary information, Fig. S2

Supplementary information, Fig. S3

Supplementary information, Fig. S4

Supplementary information, Fig. S5

Supplementary information, Fig. S6

Supplementary information, Fig. S7

Supplementary information, Fig. S8

Supplementary information, Fig. S9

Supplementary information, Fig. S10

Supplementary information, Fig. S11

Supplementary information, Fig. S12

Supplementary information, Fig. S13

Supplementary information, Table S1

Supplementary information, Table S2

Supplementary information, Table S3
