# Supplementary material for: Hetero-bivalent nanobodies provide broad-spectrum protection against SARS-CoV-2 variants of concern including Omicron
Source: Cell Res. 2022 Jul 29;32(9):831–42. doi: 10.1038/s41422-022-00700-3 (PMC9334538; doi:10.1038/s41422-022-00700-3)
Supplement: Supplementary file 1 — Supplementary information, Fig. S1 [file 41422_2022_700_MOESM1_ESM.pdf]

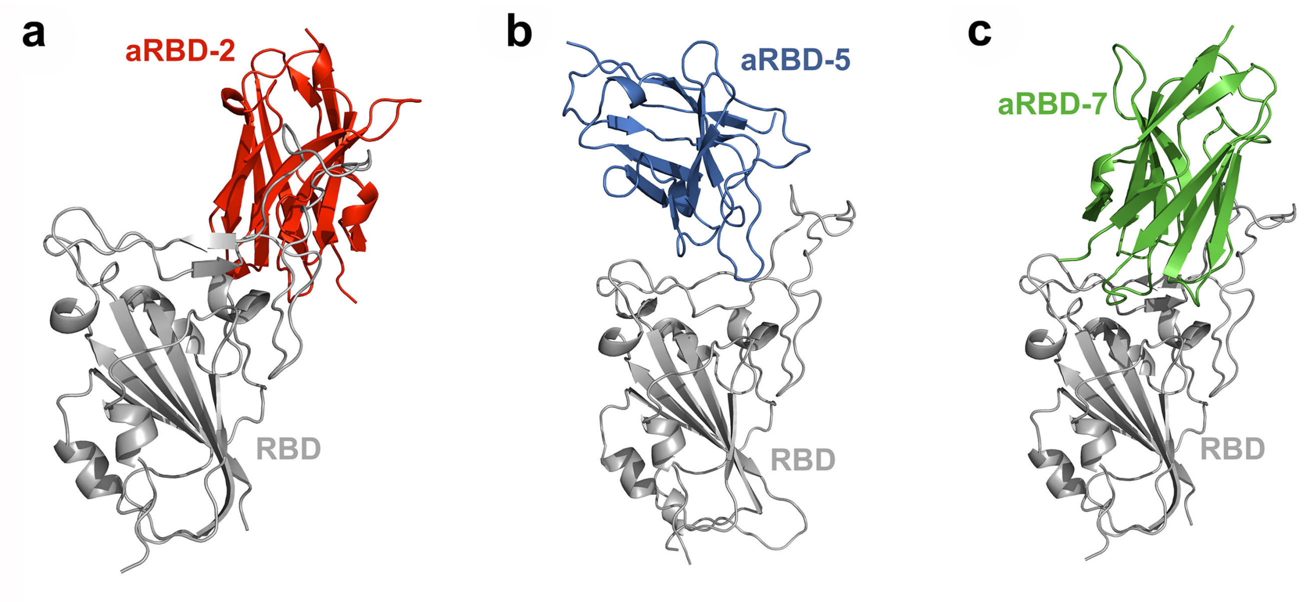

Fig. S1 Crystal structures of the descripted nanobodies in complex with RBD. aRBD-2 (**a**), aRBD-5 (**b**) and aRBD-7 (**c**) in complex with the WT SARS-CoV-2 RBD are displayed using cartoon presentation.
