# Supplementary material for: Hetero-bivalent nanobodies provide broad-spectrum protection against SARS-CoV-2 variants of concern including Omicron
Source: Cell Res. 2022 Jul 29;32(9):831–42. doi: 10.1038/s41422-022-00700-3 (PMC9334538; doi:10.1038/s41422-022-00700-3)
Supplement: Supplementary file 2 — Supplementary information, Fig. S2 [file 41422_2022_700_MOESM2_ESM.pdf]

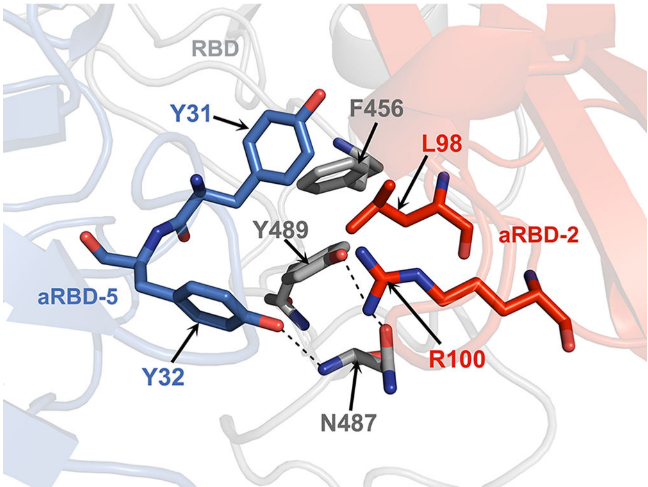


**Fig. S2 aRBD-2 and aRBD-5 share three RBD contact residues but do not clash with each other.** The structure of aRBD-2: RBD complex is superimposed on that of aRBD-5: RBD complex, and it shows that F456, N487 and Y489 of RBD interact with both aRBD-2 and aRBD-5. Specifically, the side chain of N487 and Y489 of RBD form two hydrogen bonds with the side chain of R100 of aRBD-2, while the main chain of N487 of RBD forms one hydrogen bond with the side chain of Y32 of aRBD-5, and F456 and Y489 of RBD form a hydrophobic pocket together with L98 of aRBD-2 and Y31 of aRBD-5, respectively.
