# Supplementary material for: Hetero-bivalent nanobodies provide broad-spectrum protection against SARS-CoV-2 variants of concern including Omicron
Source: Cell Res. 2022 Jul 29;32(9):831–42. doi: 10.1038/s41422-022-00700-3 (PMC9334538; doi:10.1038/s41422-022-00700-3)
Supplement: Supplementary file 3 — Supplementary information, Fig. S3 [file 41422_2022_700_MOESM3_ESM.pdf]

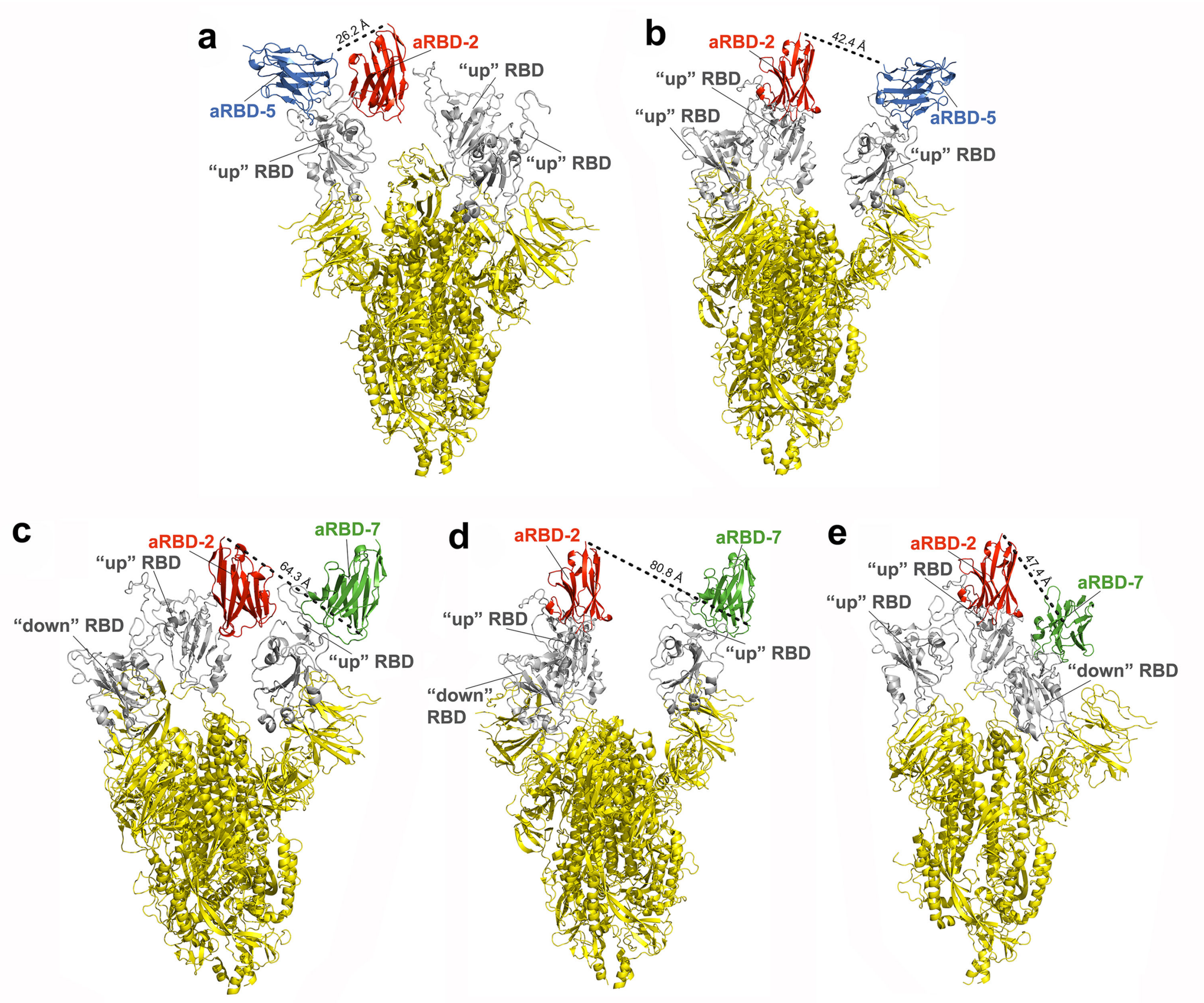

Fig. S3 The distance between the C-terminus of aRBD-2 and the N-terminus of aRBD-5 or aRBD-7 in different binding modes on the trimeric spike of SARS-CoV-2. The structures of aRBD-2 and aRBD-5 in complex with RBD were superimposed on the RBD in the cryo-EM structures of the trimeric spike with all RBD in "up" conformation (PDB: 7KMS), and the distance between the C-terminus of aRBD-2 and the N-terminus of aRBD-5 on one single RBD (**a**) or on two adjacent RBDs (**b**) was measured. The structures of aRBD-2 and aRBD-7 in complex with RBD were superimposed on the RBD in the cryo-EM structure of the trimeric spike with a two "up" and one "down" conformation (PDB: 7KMZ), and the distance between the C-terminus of aRBD-2 and the N-terminus of aRBD-7 on one single RBD (**c**), on two adjacent "up" RBDs (**d**), or on one "up" and one "down" RBDs (**e**) was measured.
