# Supplementary material for: Hetero-bivalent nanobodies provide broad-spectrum protection against SARS-CoV-2 variants of concern including Omicron
Source: Cell Res. 2022 Jul 29;32(9):831–42. doi: 10.1038/s41422-022-00700-3 (PMC9334538; doi:10.1038/s41422-022-00700-3)
Supplement: Supplementary file 4 — Supplementary information, Fig. S4 [file 41422_2022_700_MOESM4_ESM.pdf]

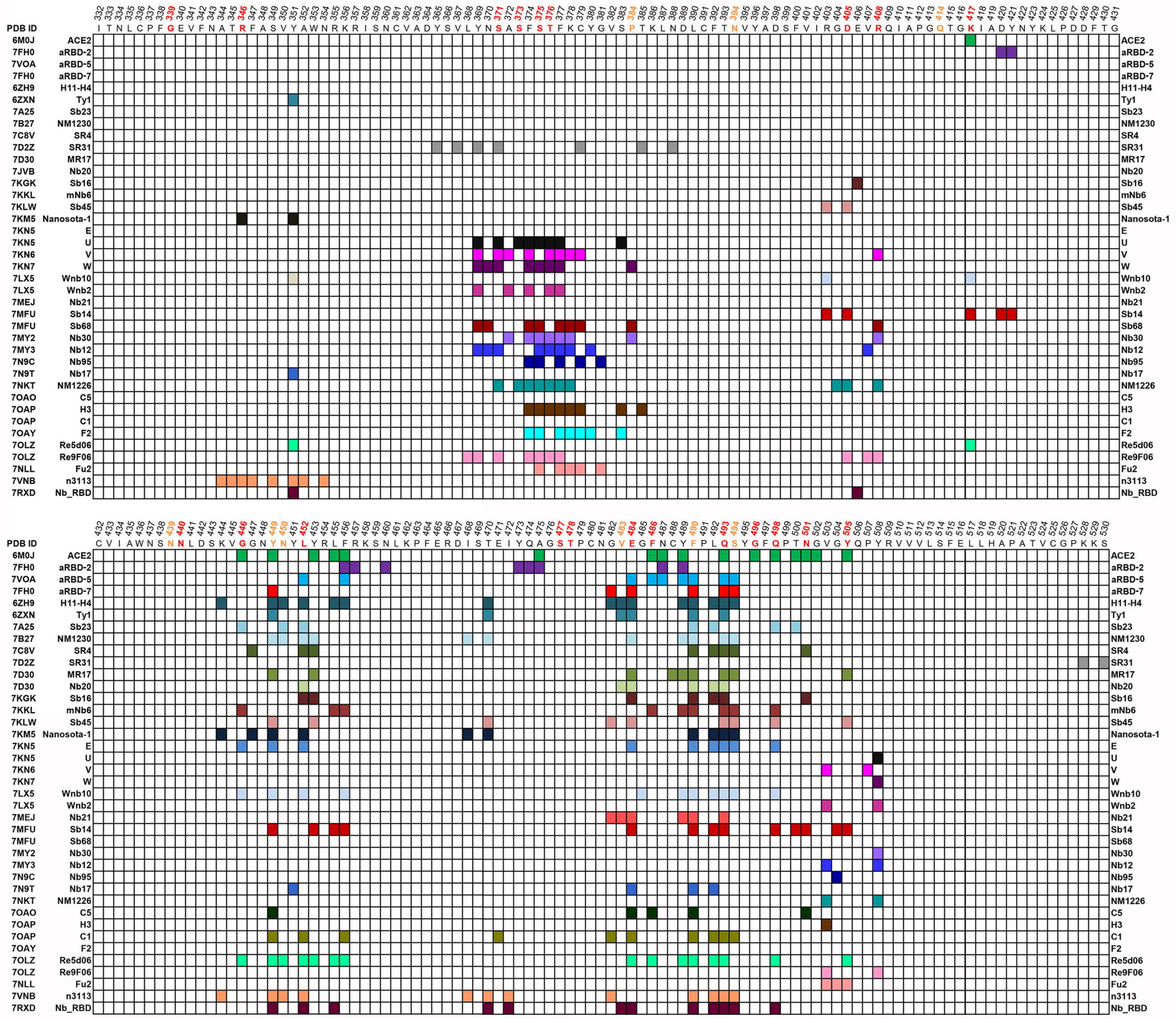

Fig. S4 The RBD residues contact with aRBD-2, aRBD-5, aRBD-7 and other previously reported Nbs. The colored squares indicate the RBD residues contact with ACE2 or each Nbs. The residues in red are mutated in Omicron BA.1, BA.1.1, BA.2, BA.3, BA.4 and BA.5, and the residues in brown are mutated in some of other VOCs, VOIs or VUMs.
