# Supplementary material for: Hetero-bivalent nanobodies provide broad-spectrum protection against SARS-CoV-2 variants of concern including Omicron
Source: Cell Res. 2022 Jul 29;32(9):831–42. doi: 10.1038/s41422-022-00700-3 (PMC9334538; doi:10.1038/s41422-022-00700-3)
Supplement: Supplementary file 6 — Supplementary information, Fig. S6 [file 41422_2022_700_MOESM6_ESM.pdf]

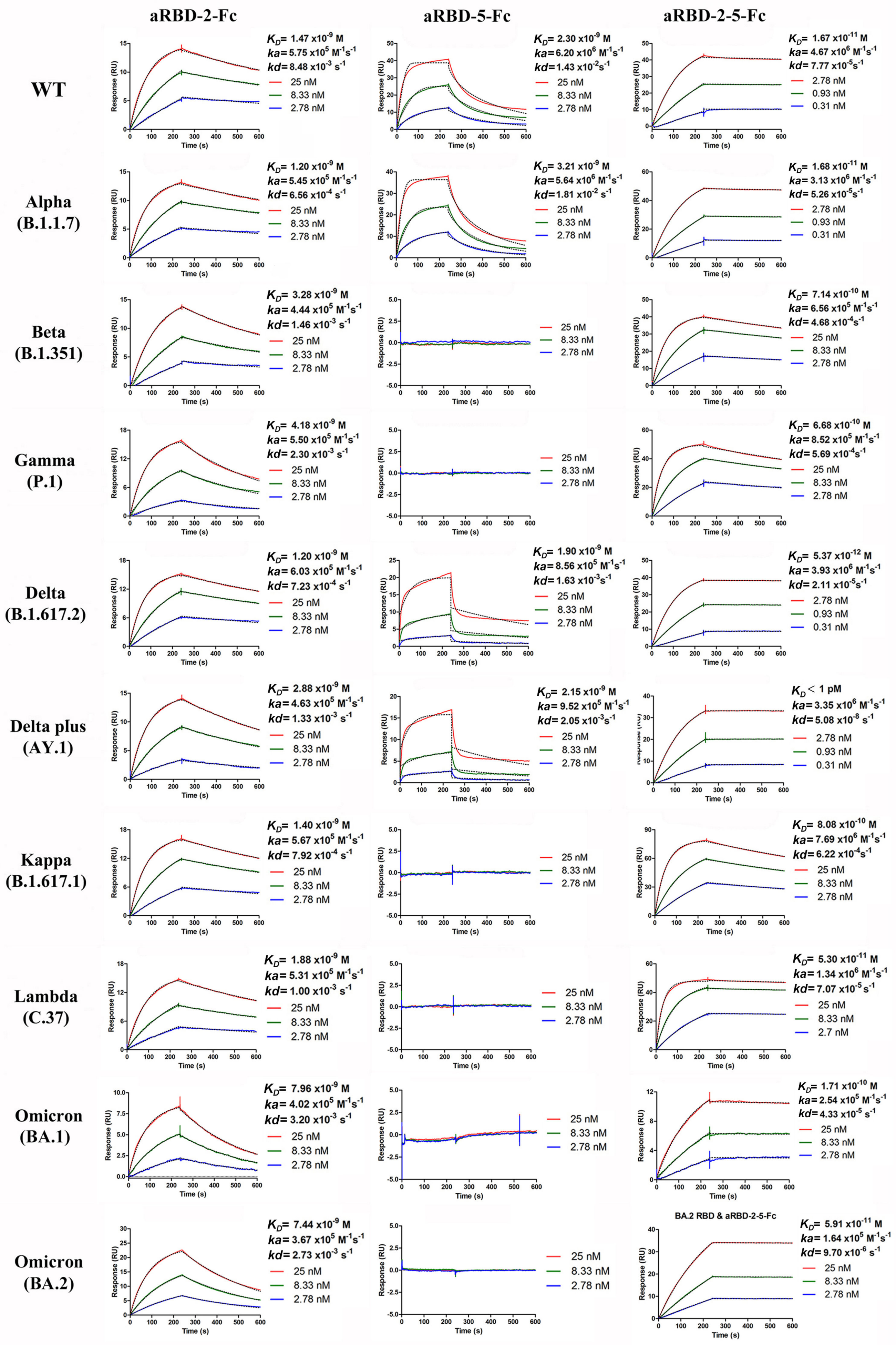

aRBD-2-Fc
aRBD-5-Fc
aRBD-2-5-Fc
WT
K_D= 1.47 x10^{-9} M
ka= 5.75 x10^{5} M^{-1}s^{-1}
kd= 8.48 x10^{-3} s^{-1}
25 nM
8.33 nM
2.78 nM
K_D= 2.30 x10^{-9} M
ka= 6.20 x10^{6} M^{-1}s^{-1}
kd= 1.43 x10^{-2} s^{-1}
25 nM
8.33 nM
2.78 nM
K_D= 1.67 x10^{-11} M
ka= 4.67 x10^{6} M^{-1}s^{-1}
kd= 7.77 x10^{-5} s^{-1}
2.78 nM
0.93 nM
0.31 nM
Alpha (B.1.1.7)
K_D= 1.20 x10^{-9} M
ka= 5.45 x10^{5} M^{-1}s^{-1}
kd= 6.56 x10^{-4} s^{-1}
25 nM
8.33 nM
2.78 nM
K_D= 3.21 x10^{-9} M
ka= 5.64 x10^{6} M^{-1}s^{-1}
kd= 1.81 x10^{-2} s^{-1}
25 nM
8.33 nM
2.78 nM
K_D= 1.68 x10^{-11} M
ka= 3.13 x10^{6} M^{-1}s^{-1}
kd= 5.26 x10^{-5} s^{-1}
2.78 nM
0.93 nM
0.31 nM
Beta (B.1.351)
K_D= 3.28 x10^{-9} M
ka= 4.44 x10^{5} M^{-1}s^{-1}
kd= 1.46 x10^{-3} s^{-1}
25 nM
8.33 nM
2.78 nM
25 nM
8.33 nM
2.78 nM
K_D= 7.14 x10^{-10} M
ka= 6.56 x10^{5} M^{-1}s^{-1}
kd= 4.68 x10^{-4} s^{-1}
25 nM
8.33 nM
2.78 nM
Gamma (P.1)
K_D= 4.18 x10^{-9} M
ka= 5.50 x10^{5} M^{-1}s^{-1}
kd= 2.30 x10^{-3} s^{-1}
25 nM
8.33 nM
2.78 nM
25 nM
8.33 nM
2.78 nM
K_D= 6.68 x10^{-10} M
ka= 8.52 x10^{5} M^{-1}s^{-1}
kd= 5.69 x10^{-4} s^{-1}
25 nM
8.33 nM
2.78 nM
Delta (B.1.617.2)
K_D= 1.20 x10^{-9} M
ka= 6.03 x10^{5} M^{-1}s^{-1}
kd= 7.23 x10^{-4} s^{-1}
25 nM
8.33 nM
2.78 nM
K_D= 1.90 x10^{-9} M
ka= 8.56 x10^{5} M^{-1}s^{-1}
kd= 1.63 x10^{-3} s^{-1}
25 nM
8.33 nM
2.78 nM
K_D= 5.37 x10^{-12} M
ka= 3.93 x10^{6} M^{-1}s^{-1}
kd= 2.11 x10^{-5} s^{-1}
2.78 nM
0.93 nM
0.31 nM
Delta plus (AY.1)
K_D= 2.88 x10^{-9} M
ka= 4.63 x10^{5} M^{-1}s^{-1}
kd= 1.33 x10^{-3} s^{-1}
25 nM
8.33 nM
2.78 nM
K_D= 2.15 x10^{-9} M
ka= 9.52 x10^{5} M^{-1}s^{-1}
kd= 2.05 x10^{-3} s^{-1}
25 nM
8.33 nM
2.78 nM
K_D < 1 pM
ka= 3.35 x10^{6} M^{-1}s^{-1}
kd= 5.08 x10^{-8} s^{-1}
2.78 nM
0.93 nM
0.31 nM
Kappa (B.1.617.1)
K_D= 1.40 x10^{-9} M
ka= 5.67 x10^{5} M^{-1}s^{-1}
kd= 7.92 x10^{-4} s^{-1}
25 nM
8.33 nM
2.78 nM
25 nM
8.33 nM
2.78 nM
K_D= 8.08 x10^{-10} M
ka= 7.69 x10^{5} M^{-1}s^{-1}
kd= 6.22 x10^{-4} s^{-1}
25 nM
8.33 nM
2.78 nM
Lambda (C.37)
K_D= 1.88 x10^{-9} M
ka= 5.31 x10^{5} M^{-1}s^{-1}
kd= 1.00 x10^{-3} s^{-1}
25 nM
8.33 nM
2.78 nM
25 nM
8.33 nM
2.78 nM
K_D= 5.30 x10^{-11} M
ka= 1.34 x10^{6} M^{-1}s^{-1}
kd= 7.07 x10^{-5} s^{-1}
25 nM
8.33 nM
2.7 nM
Omicron (BA.1)
K_D= 7.96 x10^{-9} M
ka= 4.02 x10^{5} M^{-1}s^{-1}
kd= 3.20 x10^{-3} s^{-1}
25 nM
8.33 nM
2.78 nM
25 nM
8.33 nM
2.78 nM
K_D= 1.71 x10^{-10} M
ka= 2.54 x10^{5} M^{-1}s^{-1}
kd= 4.33 x10^{-5} s^{-1}
25 nM
8.33 nM
2.78 nM
Omicron (BA.2)
K_D= 7.44 x10^{-9} M
ka= 3.67 x10^{5} M^{-1}s^{-1}
kd= 2.73 x10^{-3} s^{-1}
25 nM
8.33 nM
2.78 nM
25 nM
8.33 nM
2.78 nM
BA.2 RBD & aRBD-2-5-Fc
K_D= 5.91 x10^{-11} M
ka= 1.64 x10^{5} M^{-1}s^{-1}
kd= 9.70 x10^{-6} s^{-1}
25 nM
8.33 nM
2.78 nM
Response (RU)
Time (s)

Fig. S6 Binding affinities of aRBD-2, aRBD-5, and aRBD-2-5 for the RBD of SARS-CoV-2 variants. Binding kinetics of the RBD of SARS-CoV-2 WT, Alpha, Beta, Gamma, Delta, Delta plus, Kappa, Lambda, and Omicron variants to aRBD-2-Fc, aRBD-5-Fc, and aRBD-2-5-Fc was measured by SPR. The Nb-Fc fusions were immobilized onto a CM5 sensor chip. Serial dilutions (1:3) of RBDs were injected successively and monitored by the Biacore T200 system. The actual responses (colored lines) and the data fitted to a 1:1 binding model (black dotted lines) are shown. K_D, the equilibrium dissociation constant; *ka*, the association constant; *kd*, the dissociation constant.
