# Supplementary material for: Hetero-bivalent nanobodies provide broad-spectrum protection against SARS-CoV-2 variants of concern including Omicron
Source: Cell Res. 2022 Jul 29;32(9):831–42. doi: 10.1038/s41422-022-00700-3 (PMC9334538; doi:10.1038/s41422-022-00700-3)
Supplement: Supplementary file 7 — Supplementary information, Fig. S7 [file 41422_2022_700_MOESM7_ESM.pdf]

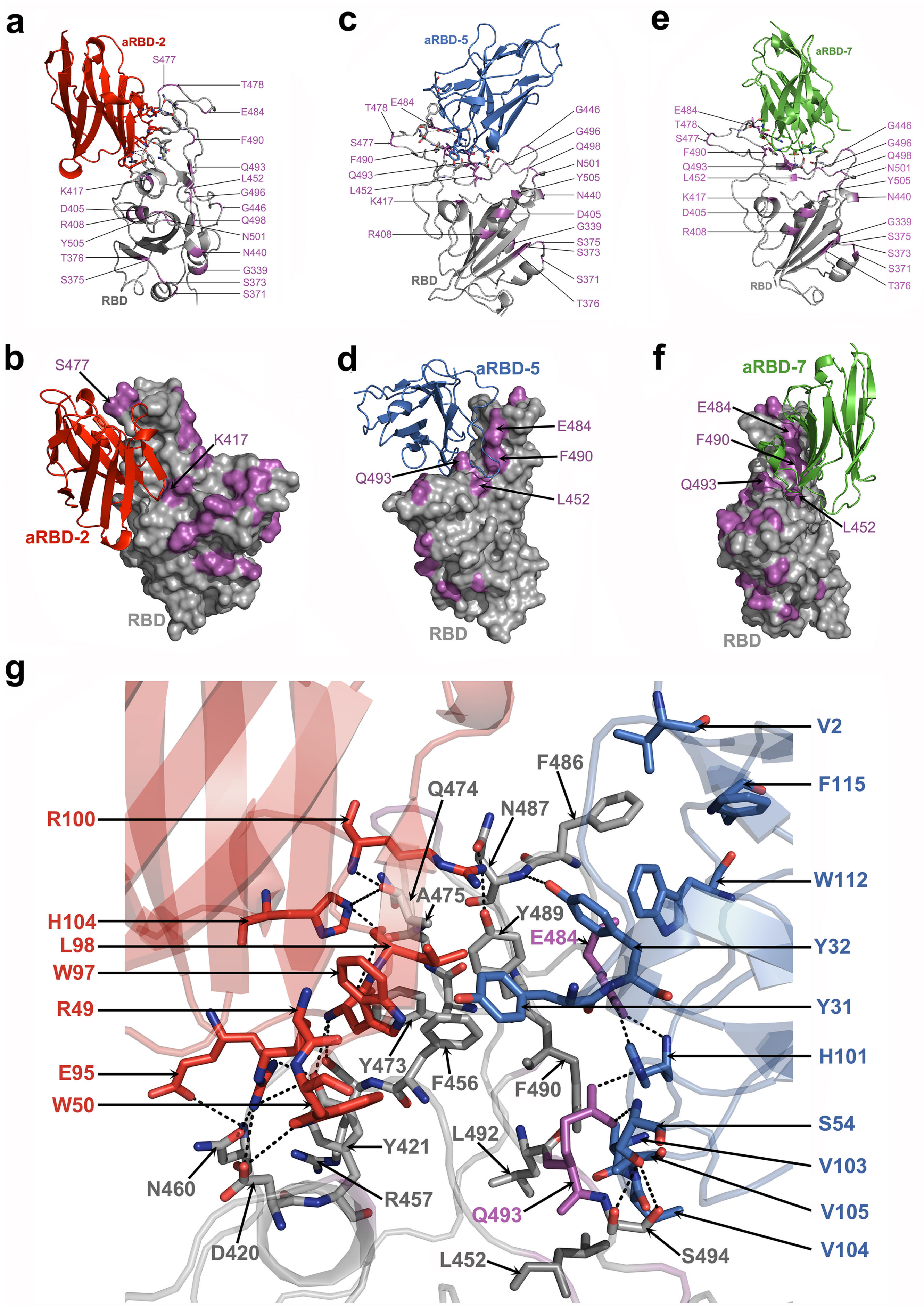

a
aRBD-2
S477
T478
E484
F490
Q493
L452
G496
G446
Q498
N501
N440
G339
S373
S371
K417
D405
R408
Y505
T376
S375
RBD
c
aRBD-5
T478
E484
S477
F490
Q493
L452
K417
R408
G446
G496
Q498
N501
Y505
N440
D405
G339
S375
S373
S371
T376
RBD
e
aRBD-7
E484
T478
S477
F490
Q493
L452
K417
D405
R408
G446
G496
Q498
N501
Y505
N440
G339
S375
S373
S371
T376
RBD
b
S477
K417
aRBD-2
RBD
d
aRBD-5
E484
F490
Q493
L452
RBD
f
aRBD-7
E484
F490
Q493
L452
RBD
g
R100
H104
L98
W97
R49
E95
W50
Q474
A475
N487
F486
Y489
E484
Y473
F456
F490
Y421
R457
N460
D420
L492
Q493
L452
S494
V2
F115
W112
Y32
Y31
H101
S54
V103
V105
V104

Fig. S7 Display of mutation sites that affect the RBD binding of aRBD-2, aRBD-5, and aRBD-7. A total of 20 sites (magenta), including G339, S371, S373, S375, T376, D405, R408, K417, N440, G446, L452, S477, T478, E484, F490, Q493, G496, Q498, N501 and Y505, are mutated in the RBD of Alpha, Beta, Gamma, Delta, Delta plus, Kappa, Lambda, Omicron BA.1 and BA.2 variants. These mutation sites are displayed on the structures of aRBD-2: RBD (**a**), aRBD-5: RBD (**c**), and aRBD-7: RBD (**e**) complexes. Residues that form interactions are shown as sticks, and hydrogen bonds and salt bridges between the Nbs and RBD are shown as black dotted lines. Residues that may affect the RBD binding of the aRBD-2 (**b**), aRBD-5 (**d**) and aRBD-7 (**f**) are indicated by arrows. **g** Possible mechanism by which aRBD-5 contributes to the overall binding affinity of aRBD-2-5 for the Omicron RBD. The structure of aRBD-2: RBD complex is aligned with that of aRBD-5: RBD complex, interacting residues are shown as sticks, and hydrogen bonds and salt bridges between the Nbs and RBD are shown as black dotted lines. E484 and Q493 in magenta are two mutation sites in the Omicron variant.
