# Supplementary material for: Hetero-bivalent nanobodies provide broad-spectrum protection against SARS-CoV-2 variants of concern including Omicron
Source: Cell Res. 2022 Jul 29;32(9):831–42. doi: 10.1038/s41422-022-00700-3 (PMC9334538; doi:10.1038/s41422-022-00700-3)
Supplement: Supplementary file 8 — Supplementary information, Fig. S8 [file 41422_2022_700_MOESM8_ESM.pdf]

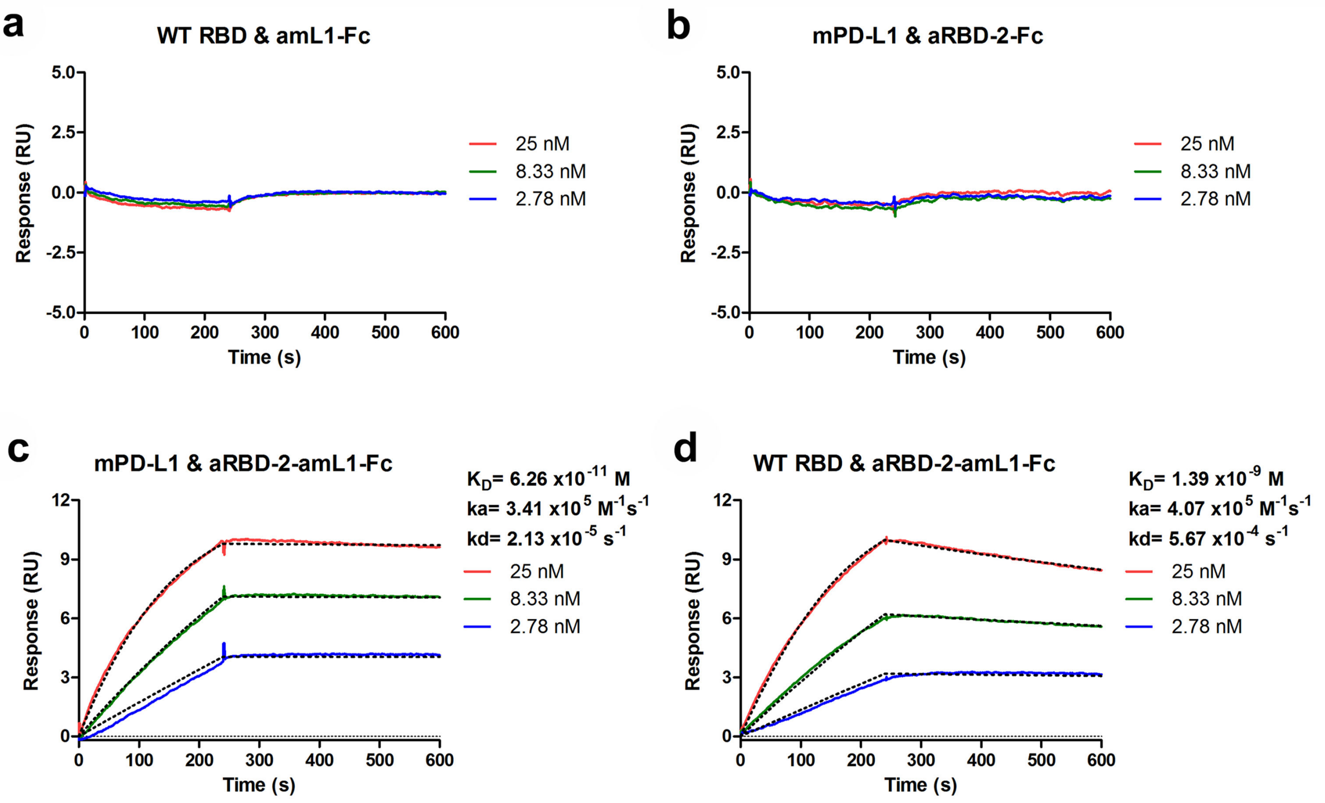

Fig. S8 RBD-binding characterization of a fusion constructed by fusing aRBD-2 to a RBD unrelated Nb. aRBD-2 was fused to a RBD unrelated Nb (an anti-mouse PD-L1 nanobody, PDB ID: 5DXW, named as amL1) and the human IgG1 Fc to form a fusion termed as aRBD-2-amL1-Fc. SPR showed that no binding were detected between the amL1-Fc and the WT SARS-CoV-2 RBD (**a**), and between aRBD-2 and the mouse PD-L1 (**b**). The aRBD-2-amL1-Fc fusion showed binding to both the mouse PD-L1 (**c**) and the RBD (**d**). The RBD binding affinity K_D of aRBD-2-amL1-Fc is 1.39 nM, which is similar to that of aRBD-2-Fc (K_D = 1.47 nM).
