# Supplementary material for: Hetero-bivalent nanobodies provide broad-spectrum protection against SARS-CoV-2 variants of concern including Omicron
Source: Cell Res. 2022 Jul 29;32(9):831–42. doi: 10.1038/s41422-022-00700-3 (PMC9334538; doi:10.1038/s41422-022-00700-3)
Supplement: Supplementary file 9 — Supplementary information, Fig. S9 [file 41422_2022_700_MOESM9_ESM.pdf]

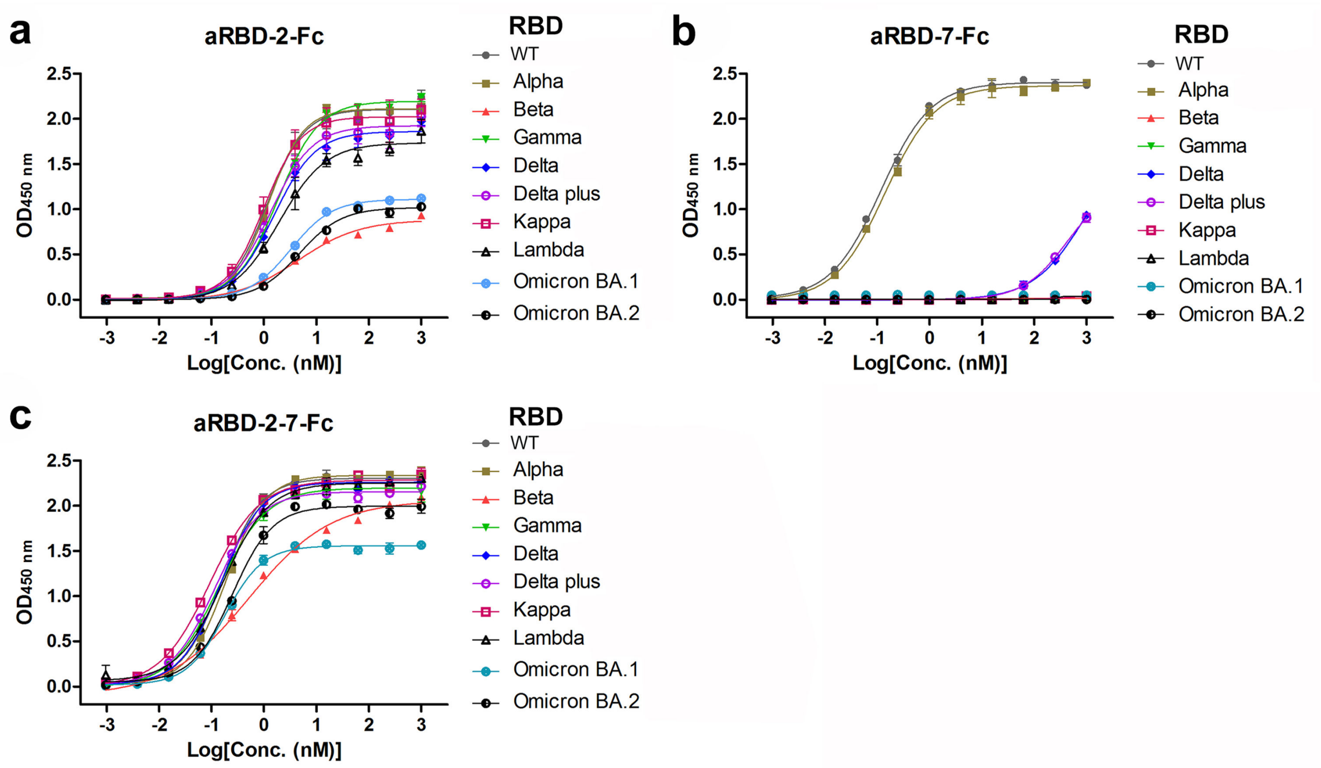

Fig. S9 Binding characterization of aRBD-2, aRBD-7, and aRBD-2-7 to the RBDs of SARS-CoV-2 variants using ELISA. aRBD-2-Fc (**a**), aRBD-7-Fc (**b**), and aRBD-2-7-Fc (**c**) binding to the RBDs of SARS-CoV-2 variants were characterized using ELISA. EC_{50} were calculated by fitting the OD_{450} values from the serially diluted antibody to a sigmoidal dose-response curve. Error bars indicate mean ± standard deviation (SD) from two independent experiments.
