# Supplementary material for: Hetero-bivalent nanobodies provide broad-spectrum protection against SARS-CoV-2 variants of concern including Omicron
Source: Cell Res. 2022 Jul 29;32(9):831–42. doi: 10.1038/s41422-022-00700-3 (PMC9334538; doi:10.1038/s41422-022-00700-3)
Supplement: Supplementary file 10 — Supplementary information, Fig. S10 [file 41422_2022_700_MOESM10_ESM.pdf]

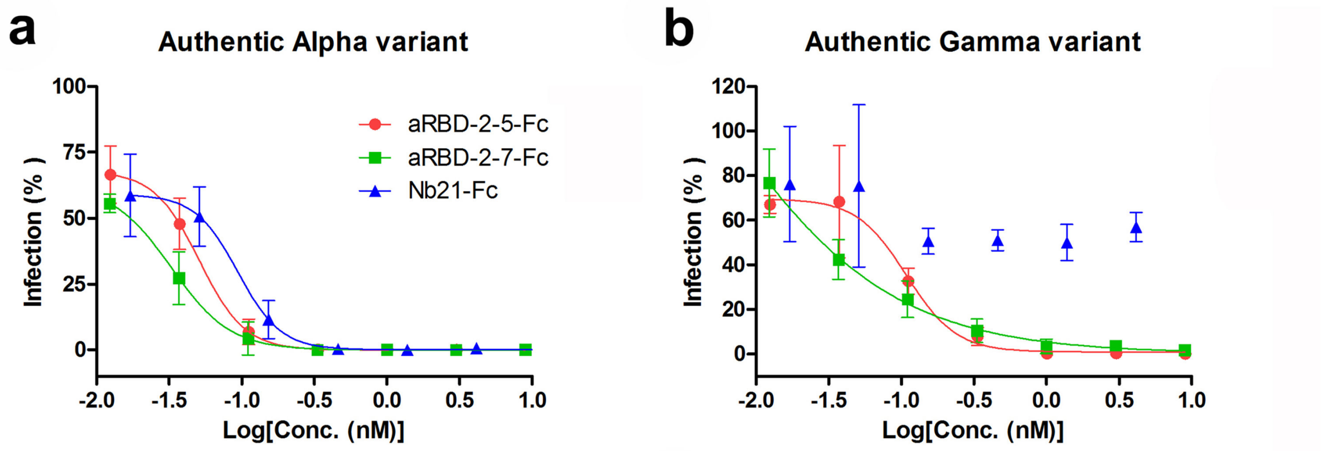

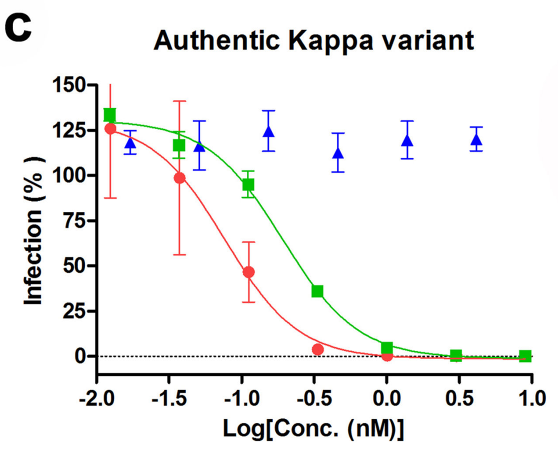

d

IC50 (nM) Virus / Antibodies	Alpha	Gamma	Kappa
aRBD-2-5-Fc	0.0511	0.1087	0.0769
aRBD-2-7-Fc	0.0328	0.0045	0.1914
Nb21-Fc	0.0943	ND*	ND

Fig. S10 Neutralization of authentic Alpha, Gamma and Kappa variants by aRBD-2-5-Fc and aRBD-2-7-Fc using microneutralization assay. Approximately 200 PFU of authentic Alpha (**a**), Gamma (**b**), and Kappa (**c**) variants were neutralized with serially diluted antibodies. Error bars indicate the mean ± SD from three independent experiments. IC_{50} values were calculated by fitting the Infection (%) values of the serial dilution with a sigmoidal dose-response curve, and listed in (**d**). *ND, neutralization not detected.
