# Supplementary material for: Hetero-bivalent nanobodies provide broad-spectrum protection against SARS-CoV-2 variants of concern including Omicron
Source: Cell Res. 2022 Jul 29;32(9):831–42. doi: 10.1038/s41422-022-00700-3 (PMC9334538; doi:10.1038/s41422-022-00700-3)
Supplement: Supplementary file 11 — Supplementary information, Fig. S11 [file 41422_2022_700_MOESM11_ESM.pdf]

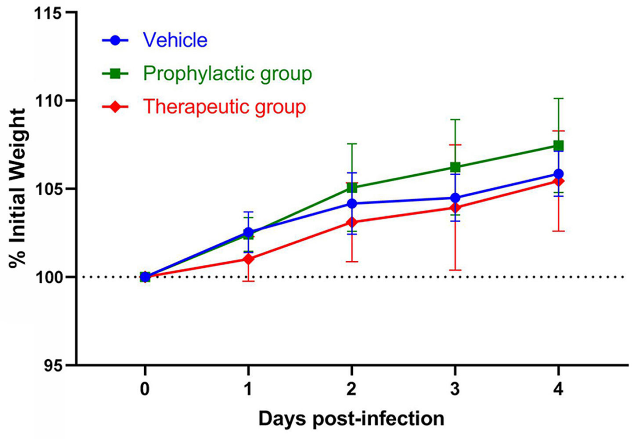

Fig. S11 Body weight changes of treated hamsters. Body weight changes of hamsters treated with aRBD-2-5-Fc prophylactically and therapeutically or PBS.
