# Supplementary material for: Hetero-bivalent nanobodies provide broad-spectrum protection against SARS-CoV-2 variants of concern including Omicron
Source: Cell Res. 2022 Jul 29;32(9):831–42. doi: 10.1038/s41422-022-00700-3 (PMC9334538; doi:10.1038/s41422-022-00700-3)
Supplement: Supplementary file 12 — Supplementary information, Fig. S12 [file 41422_2022_700_MOESM12_ESM.pdf]

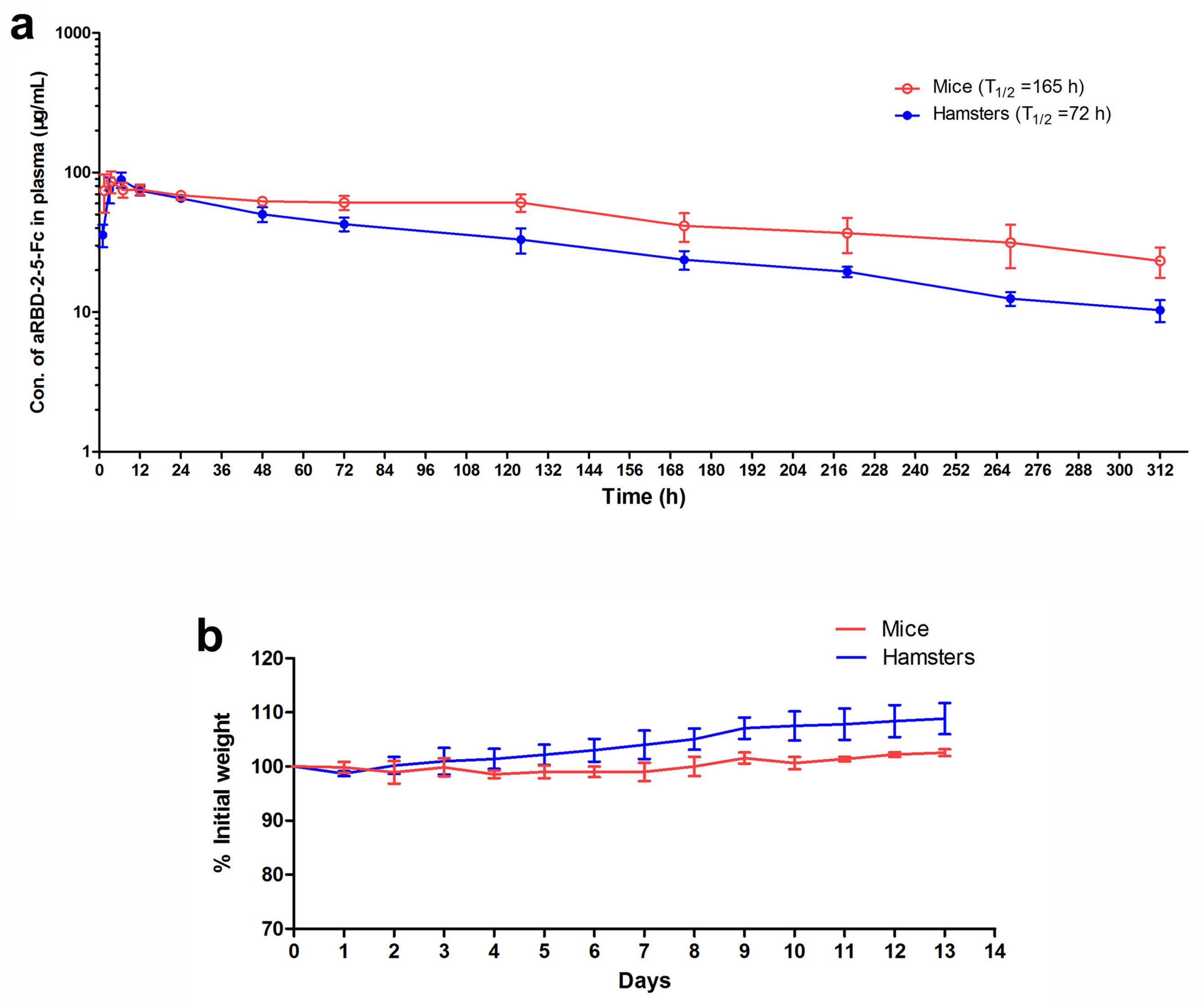

Fig. S12 The half-life of aRBD-2-5-Fc in mice and hamsters. C57BL/6 mice (n = 4) and Syrian golden hamsters (n = 3) were i.p. administered with aRBD-2-5-Fc at single dose of 10 mg/kg. **a** The animal blood was collected at different time points from 1 to 312 h after administration, and the aRBD-2-5-Fc concentration in plasma was detected by ELISA.
b Body weight changes of the animals after administration. Error bars indicate mean ± SD.
