# Supplementary material for: Hetero-bivalent nanobodies provide broad-spectrum protection against SARS-CoV-2 variants of concern including Omicron
Source: Cell Res. 2022 Jul 29;32(9):831–42. doi: 10.1038/s41422-022-00700-3 (PMC9334538; doi:10.1038/s41422-022-00700-3)
Supplement: Supplementary file 13 — Supplementary information, Fig. S13 [file 41422_2022_700_MOESM13_ESM.pdf]

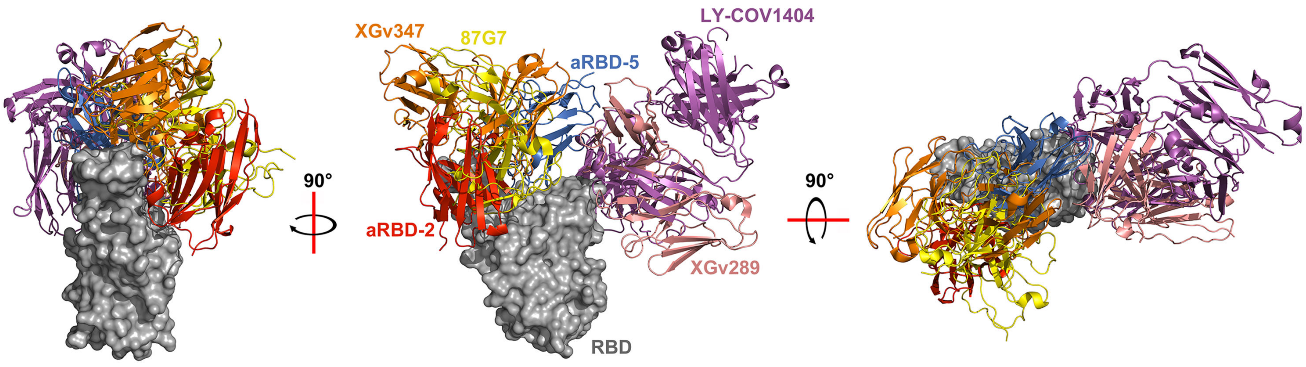

Fig. S13 Structural alignment of aRBD-2-5 and other Omicron neutralizing antibodies. Superimposition of aRBD-2 (red), aRBD-5 (marine) and conventional potent Omicron BA.1/BA.2 neutralizing antibodies, including LY-COV1404 (purple, PDB ID: 7MMO), XGv347 (orange, PDB ID: 7WEA), XGv289 (salmon, PDB ID: 7WE9) and 87G7 (yellow, PDB ID: 7R40) onto WT RBD.
