# Supplementary material for: Hetero-bivalent nanobodies provide broad-spectrum protection against SARS-CoV-2 variants of concern including Omicron
Source: Cell Res. 2022 Jul 29;32(9):831–42. doi: 10.1038/s41422-022-00700-3 (PMC9334538; doi:10.1038/s41422-022-00700-3)
Supplement: Supplementary file 14 — Supplementary information, Table S1 [file 41422_2022_700_MOESM14_ESM.pdf]

Table S1. Data collection and refinement statistics

	RBD-tr2: aRBD-2-7	RBD: aRBD-5
Data collection	BL02U1	BL19U1
Wavelength (Å)	0.97918	0.97852
Space group	$P6_522$	$P2_12_12_1$
Unit cell parameters		
a, b, c (Å)	154.46, 154.46, 257.92	53.44, 87.70, 89.06
α, β, γ (°)	90, 90, 120	90, 90, 90
Resolution range (Å)	50.00-3.20 (3.37-3.20)	45.82-1.80 (1.85-1.80)
Monomers in an asymmetric unit	2	1
Unique reflections	30769 (4397)	38650 (2755)
Average redundancy	18.5 (19.0)	12.9 (12.4)
Completeness (%)	100 (100)	97.8 (95.4)
R_{merge} (%)[a]	15.3 (126.1)	10.2 (242.4)
I/σ (I)	20.3 (3.2)	13.14 (1.17)
Wilson B factor (Å^2)	84.6	33.1
Refinement Statistics		
Resolution range (Å)	50.00-3.20	45.82-1.80
R_{work} (%)[b]	20.62	18.63
R_{free} (%)[c]	26.22	22.62
RMSD bond lengths (Å)	0.0078	0.016
RMSD bond angles (°)	1.2814	1.370
Mean B factors (Å^2)		
Protein	97.58	48.96
Ligand	136.82	78.43
Water	72.62	52.18
No. of non-hydrogen protein atoms	6945	2536
No. of ligand atoms	75	14
No. of water oxygen atoms	56	175
Ramachandran plot[d]		
Favored (%)	96.0	96.94
Outliers (%)	0	0.31
PDB entry	7FH0	7VOA

[a]$R_{merge} = \sum_{hkl}\sum_i |I_i(hkl) - \langle I(hkl)\rangle| / \sum_{hkl}\sum_i I_i(hkl)$, where $I_i(hkl)$ is the intensity of *ith* observation and <I(hkl)> is the mean value for reflection hkl.

[b]$R_{work}=\sum_{hkl}\big||F_{obs}|-|F_{cacl}|\big|/\sum_{hkl}|F_{obs}|$, where F_{obs} and F_{calc} are the observed and calculated structure-factor amplitudes, respectively.

[c]R_{free} is calculated same as R_{work} with 5% reflections, which were selected randomly from the refinement process.

[d]The categories were defined by PROCHECK. The numbers in brackets are the parameters for the “Resolution range,” “Unique reflection,” “Average redundancy,” “Completeness,” “R_{merge},”and “I/σ(I)”of the highest resolution shell.
