# Supplementary material for: Hetero-bivalent nanobodies provide broad-spectrum protection against SARS-CoV-2 variants of concern including Omicron
Source: Cell Res. 2022 Jul 29;32(9):831–42. doi: 10.1038/s41422-022-00700-3 (PMC9334538; doi:10.1038/s41422-022-00700-3)
Supplement: Supplementary file 15 — Supplementary information, Table S2 [file 41422_2022_700_MOESM15_ESM.pdf]

Table S2. Interactions between the Nbs and SARS-CoV-2 RBD.

RBD		aRBD-2		Bond
Residue	atom	Residue	atom	
D420	OD2	R49	NH1	Hydrogen bond
D420	OD2	W50	NE1	Hydrogen bond
Y421	OH	R49	NH1	Salt bridge
Y421	OH	W97	N	Hydrogen bond
Y421	OH	E95	O	Hydrogen bond
Y421		R49		Cation-π
R457	O	W97	N	Hydrogen bond
N460	N	E95	OE1	Hydrogen bond
N460	OD1	R49	NH2	Hydrogen bond
Y473	OH	L98	O	Hydrogen bond
Q474	O	H104	NE2	Hydrogen bond
A475	O	R100	N	Hydrogen bond
A475	O	H104	NE2	Hydrogen bond
N487	OD1	R100	NH1	Hydrogen bond
Y489	OH	R100	NH1	Hydrogen bond
F456/Y489		L98		Hydrophobic interaction

RBD		aRBD-5		Bond
Residue	atom	Residue	atom	
F456,Y489		Y31		Hydrophobic interaction
E484	OE1	H101	N	Hydrogen bond
E484	OE2	H101	ND1	Hydrogen bond
F486		V2, W112,F115		Hydrophobic interaction
N487	N	Y32	OH	Hydrogen bond
Q493	OE1	S54	N	Hydrogen bond
Q493	NE2	H101	NE2	Hydrogen bond
S494	O	S54	OG	Hydrogen bond
S494	OG	S54	OG	Hydrogen bond
L452, F490 and L492		V103, V104 and A105		Hydrophobic interaction

RBD		aRBD-7		Bond
Residue	atom	Residue	atom	
Y449	OH	R101	NH1	Salt bridge
G482	O	T58	N	Hydrogen bond
E484	OE1	R52	NH2	Hydrogen bond
E484	OE2	R52	NE	Hydrogen bond
E484	OE2	R52	NH2	Salt bridge
E484	OE1	S57	OG	Hydrogen bond
E484	N	T58	O	Hydrogen bond

F490		R52		Cation-π
F490	N	A104	O	Hydrogen bond
Q493	OE1	A104	N	Hydrogen bond
S494	OG	T102	N	Hydrogen bond
S494	N	T102	O	Hydrogen bond
