# Supplementary material for: Hetero-bivalent nanobodies provide broad-spectrum protection against SARS-CoV-2 variants of concern including Omicron
Source: Cell Res. 2022 Jul 29;32(9):831–42. doi: 10.1038/s41422-022-00700-3 (PMC9334538; doi:10.1038/s41422-022-00700-3)
Supplement: Supplementary file 16 — Supplementary information, Table S3 [file 41422_2022_700_MOESM16_ESM.pdf]

Table S3. Number of sequences containing a single mutation in GISAID EpiCoV database

Position in RBD	Residues	Number of the sequence
D420	A	16
D420	D	5971205
D420	E	6
D420	G	24
D420	N	56
D420	V	6
D420	Y	18
Y421	C	6
Y421	F	153
Y421	H	2
Y421	N	2
Y421	S	2
Y421	Y	5971166
F456	C	6
F456	F	5970563
F456	L	689
F456	S	6
F456	V	67
R457	K	12
R457	R	5971279
R457	S	40
N460	-*	2
N460	D	146
N460	H	10
N460	I	44
N460	K	217
N460	N	5969316
N460	S	1277
N460	T	56
N460	Y	263
Y473	-	29
Y473	F	30
Y473	H	44
Y473	N	2
Y473	Y	5971226
Q474	-	37
Q474	E	48
Q474	H	323
Q474	K	18
Q474	L	1
Q474	P	10
Q474	Q	5970854

Q474	R	40
A475	-	51
A475	A	5970174
A475	D	4
A475	G	2
A475	P	6
A475	S	369
A475	T	77
A475	V	648
N487	-	73
N487	D	2
N487	K	2
N487	N	5971250
N487	S	2
N487	Y	2
Y489	-	81
Y489	F	6
Y489	H	65
Y489	N	1
Y489	Y	5971178
E484	-	77
E484	A	2183983
E484	D	231
E484	E	3762809
E484	G	289
E484	H	2
E484	K	8578
E484	P	99
E484	Q	13508
E484	R	9
E484	S	87
E484	T	62
E484	V	1597
N501	-	121
N501	C	2
N501	E	2
N501	F	4
N501	H	8
N501	I	167
N501	K	14
N501	L	4
N501	M	6
N501	N	3779640
N501	P	2

N501	S	555
N501	T	809
N501	Y	2189997

*-, residue deletion. E484 and N501 are residues used as analysis controls.
