# Supplementary material for: Hetero-bivalent nanobodies provide broad-spectrum protection against SARS-CoV-2 variants of concern including Omicron
Source: Cell Res. 2022 Jul 29;32(9):831–42. doi: 10.1038/s41422-022-00700-3 (PMC9334538; doi:10.1038/s41422-022-00700-3)
Supplement: Supplementary file 5 — Supplementary information, Fig. S5 [file 41422_2022_700_MOESM5_ESM.pdf]

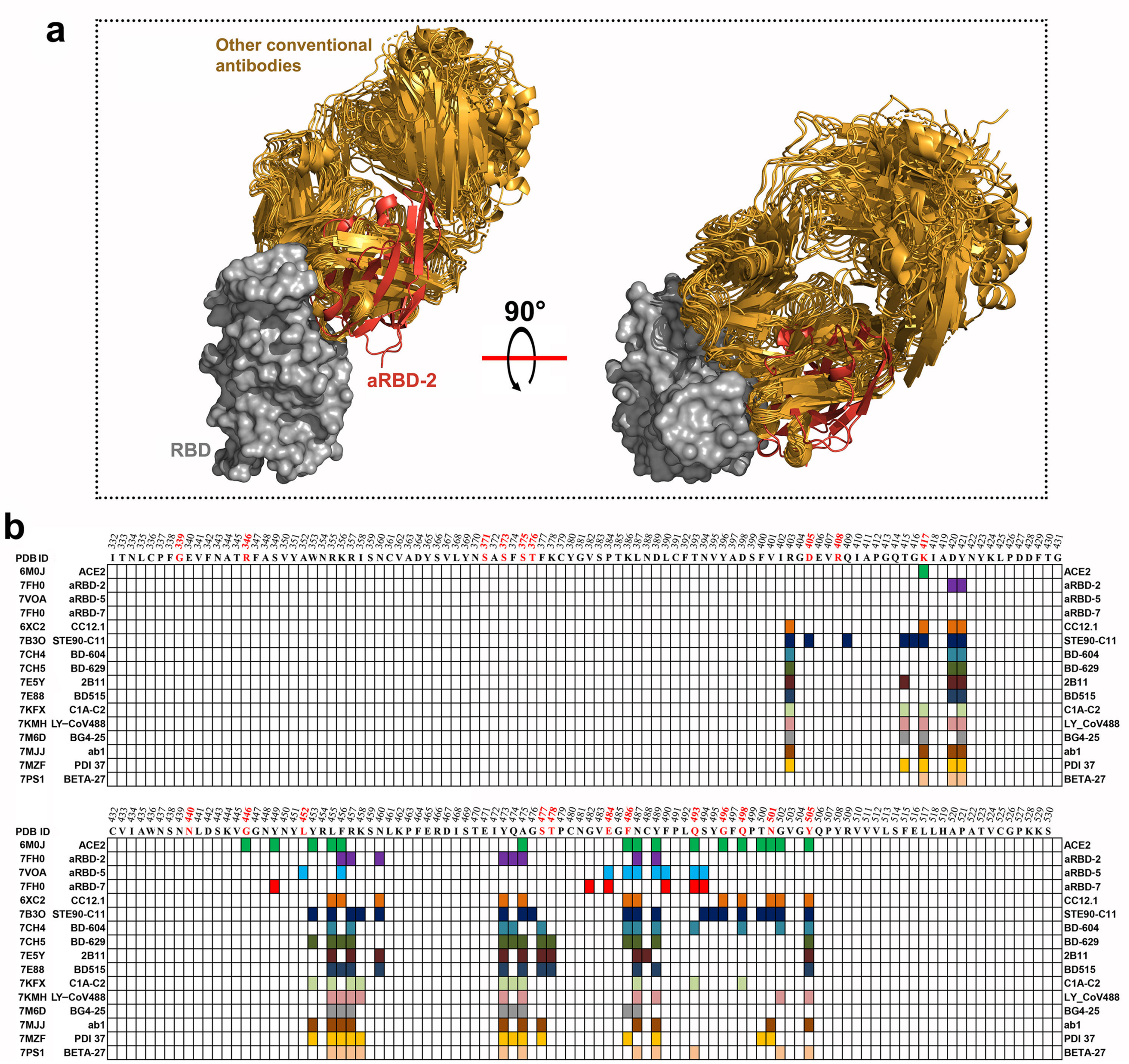

Fig. S5 Epitope comparison between aRBD-2 and some conventional antibodies that cover the aRBD-2 binding epitope. a The structural alignment of aRBD-2 bound to RBD and the conventional antibodies bound to RBD. **b** The comparison of RBD contact residues. The colored squares indicate the RBD residues contact with ACE2, our Nbs and the conventional antibodies, and the residues in red are mutated in Omicron BA.1, BA.1.1, BA.2, BA.3, BA.4 and BA.5.
